# Is functional training functional? a systematic review of its effects in community-dwelling older adults

**DOI:** 10.1186/s11556-024-00366-3

**Published:** 2024-12-21

**Authors:** Chiung-ju Liu, Wen-Pin Chang, Yun Chan Shin, Yi-Ling Hu, Jane Morgan-Daniel

**Affiliations:** 1 Department of Occupational Therapy, College of Public Health and Health Professions, University of Florida, 1225 Center Drive, P.O. Box 100164, Gainesville, FL 32610-0164 USA; 2 Department of Occupational Therapy, College of Health Professions, University of Texas Rio Grande Valley, Edinburg, TX USA; 3 Department of Occupational Therapy, Chang Gung University, Taoyuan, Taiwan; 4 Health Science Center Libraries, University of Florida, Gainesville, USA

**Keywords:** Activities of daily living, Cognitive impairment, Dementia, Disability, Functional training, Frailty, Physical functioning

## Abstract

**Background:**

Age-related decline in physical and cognitive capacity increases older adults’ risk of disability, long-term care placement, and mortality rate. Functional training, which uses activities of daily living or simulated movements to complete activities as the intervention medium, could be more effective than rote exercise, which uses repetitive movements without added purpose, in preventing late-life disability in older people. With a growing number of studies in this area, systematically studying the effect of functional training is needed. The purpose of this systematic review was to examine the effects of functional training on the outcomes of activities of daily living, physical functioning, and cognitive function in community-dwelling older adults.

**Methods:**

Literature published between January 2010 and April 2024 in 10 electronic databases were searched and screened. This timeframe was established to include studies published within the last 15 years. Each identified article was screened and reviewed by two authors independently. The methodological quality of the included studies was evaluated using the PEDro Scale. Key findings were synthesized according to participants’ characteristics and intervention types.

**Results:**

The review included 32 studies. In the general community-dwelling older adult population (20 studies), studies that applied functional training as a single-component approach showed a positive effect on activities of daily living. However, the training effect on balance and mobility was not superior to that of other exercise programs. Moreover, the effect was mixed when functional training was combined with other intervention components. In older adults with mild cognitive impairment (5 studies), Simulated Functional Tasks Exercise, a single-component training, consistently demonstrated positive effects on the activities of daily living and cognitive functions. In older adults with dementia (4 studies) or frailty (3 studies), the effect was mixed across the single- and multi-component approaches.

**Conclusion:**

Functional training alone is effective in preventing late-life disability in general community-dwelling older adults. When training activities challenge both motor and cognitive abilities, the effect seems to improve the performance of activities of daily living and cognitive functions in older adults with mild cognitive impairment. Additional studies of functional training in older adults with cognitive impairment or frailty are recommended.

**Supplementary Information:**

The online version contains supplementary material available at 10.1186/s11556-024-00366-3.

## Background

Maintaining older adults’ ability to take care of themselves and live independently is a top public health priority due to a significant increase in life expectancy over the past few decades [1]. Age-related physical or cognitive decline increases older adults’ risk of becoming care dependent. The risk of care dependency is higher for older adults who are frail or have cognitive impairment than for those without frailty or cognitive impairment [2, 3]. Care dependency imposes substantial costs and burdens on individuals, families, and society [4, 5].

Physical exercise is commonly recommended to support healthy aging [6, 7]. Countless clinical trials and systematic reviews have repeatedly examined the physical and cognitive benefits of physical exercise for older adults [8–11]. While these studies generally affirm the positive benefits of late-life exercise programs, the extent to which these benefits translate to the ability to perform activities of daily living (ADLs) and instrumental activities of daily living (IADLs), or the ability to live independently is not entirely certain [10–12]. Examining how different types of exercise may benefit older adults’ ability to perform ADLs remains a primary interest of researchers. One type of exercise is functional training.

Functional training involves daily movement patterns and requires the synergistic effects of different physical capacities, such as muscle strength and balance [13, 14]. The key principle of functional training is specificity [15], meaning that people become better at what the training specifically targets. Functional training has been applied to help athletes integrate various physical capabilities to improve sports performance [16]. Functional training has also been applied to help older adults improve their physical functioning and ADL performance. A systematic review of 13 studies in 2014 showed that functional training improved older adults’ mobility and reduced their ADL disability [14]. However, the training content in these studies was heterogeneous, ranging from movement-specific exercises, such as practicing stair climbing, to activity-oriented tasks, such as performing laundry or vacuuming activities. Despite this heterogeneity, functional training primarily addressed physical decline in prior research.

Recently, interest in applying functional training to older adults with cognitive impairment has grown [17–19]. Unlike rote exercise, which refers to the mechanical or unthinking repetition of movements without added purpose or imagery (e.g., kicking without a ball), functional training involves more complex movements and motor planning, which can challenge cognitive abilities. By mimicking daily movement patterns and tasks, functional training may be more meaningful and practical for older adults with cognitive impairment.

This systematic review aimed to build upon the previous examination of functional training [14] by including frail older adults and older adults with cognitive impairment. Additionally, the current review aimed to explore the effect of training on cognitive functions. By assessing cognitive outcomes associated with functional training, this review could shed light on how functional training affects cognition and offers practical strategies for preserving cognitive function and potentially delaying further decline in older adults. The review question was: What are the effects of functional training on ADLs/IADLs, physical functioning, and cognitive functions in general community-dwelling older adults, older adults with frailty, and older adults with cognitive impairment, respectively?

## Method

The PROSPERO registration record of this systemic review protocol is CRD42021271742. The PRISMA 2020 guidelines [20] were confirmed by the checklist in the Appendix.

### Literature search

A health sciences librarian developed the search terms and strategies with feedback from the review team. The search terms specified the interested populations and intervention were modified for each database using subject headings, truncation, title/abstract field searching, and phrase searching where possible. Ten databases were searched: AgeLine via EBSCOhost, CINAHL via EBSCOhost, Embase via Elsevier, PEDro, PsycINFO via EBSCOhost, Psychology and Behavioral Sciences Collection via EBSCOhost, PubMed, REHABDATA via the National Rehabilitation Information Center, SPORTDiscus via EBSCOhost, and Web of Science via Clarivate Analytics. See search terms in the Appendix. The search was limited to English language articles that were published from January 1, 2010, to the search dates (October 28, 2021; updated on January 20, 2023; and April 19, 2024).

### Literature screening

Covidence systematic review software (www.covidence.org) was used to screen and select the literature. The search results were uploaded to Covidence where automatic de-duplication of articles occurred. The literature screening process consisted of two phases: the title and abstract screening and the full-text screening. During the title and abstract screening, each record was independently reviewed by two reviewers. Discord between the two reviewers was resolved by consulting with a third reviewer or requesting a full-text review. Records were moved onto the full-text screening if they passed the initial screening or needed more information to determine eligibility. The full-text screening process was similar to that used for title and abstract screening. Reasons for exclusion at this phase were recorded.

Studies were eligible if: (1) the average age of the study sample was 60 years or older, (2) the study participants were community-dwelling, (3) functional training could be clearly identified in the intervention program, and (4) the outcome measures include physical functioning performance, cognitive functions, or ADLs/IADLs. Studies were eligible if participants with frailty or cognitive impairment were recruited. Functional training was defined as the exercise that applies movements or movement patterns used to complete daily tasks, and these movements must be more than simply walking. Studies were excluded if: (1) the participants were institutionalized or hospitalized, such as residents in skilled nursing facilities or inpatients; (2) the participants had a neurological disorder that affected motor skills, such as stroke; (3) participants aged 59 years or younger were recruited; (4) the intervention was solely muscle strengthening exercise, aerobic exercise, and/or a walking program that did not incorporate simulated movements to perform ADLs/IADLs; or (5) the study design was not a clinical trial. A clinical trial could include randomized controlled trials (RCTs) and non-randomized RCTs that prospectively assign participants to one or more groups. References in systematic review articles were screened for eligibility.

### Data extraction and synthesis

Data extraction was carried out using a standard extraction form by paired reviewers: one reviewer extracted the data, and the other verified it. Information on the research design, characteristics of the study participants, characteristics of the intervention and comparison(s), outcome measures, and findings of the statistical analysis related to ADLs/IADLs, physical functioning, and cognitive function was summarized. It was not feasible to conduct a meta-analysis due to the variability of the intervention content and outcome measures. Instead, studies were synthesized based on participant characteristics and types of functional training. Key findings regarding ADL/IADL outcomes, physical functioning, and cognitive functions were organized first by participant characteristics and then by the content of functional training. The interpretation of these findings was weighted according to the strength of evidence, which was partly determined by the research methodology quality rating.

### Assessment of methodological quality and the strength of evidence

We assessed the methodological quality of the studies included using the PEDro scale, which comprises 11 items with a maximum score of 10 [21, 22]. These 11 items are specified participant eligibility (Item 1, not scored); randomization (Item 2); concealed allocation (Item 3); compatibility between groups (Item 4); blinded participants (Item 5); blinded interventionists (Item 6); blinded assessors (Item 7); attrition (Item 8); intention to treat (Item 9); between-group comparison results (Item 10); and treatment effect and measures of variability for at least one key outcome (Item 11). The first item was excluded from the total score calculation. A higher score on the scale indicates greater methodological quality or internal validity.

The PEDro Scale has demonstrated good reliability and validity in assessing the methodological quality of clinical therapy intervention studies [23–25]. The scale was appropriate and reliable for rating non-RCTs and RCTs [26, 27]. For non-RCTs, inapplicable items were scored as “NA.” For example, Items 2, 3, 4, and 10 were automatically scored as “NA” for the one-group pre-posttest design. Two independent reviewers assessed each study, and the final consensus score was reported.

The strength of evidence was rated as high, moderate, or low according to the level of certainty [28]. High strength of evidence indicates that the available evidence includes consistent results from well-designed, well-conducted studies and is unlikely to be strongly affected by the results of future studies. Well-conducted studies are indicated by high PEDro scores. Moderate strength of evidence indicates that the available evidence is sufficient to determine the effects; however, confidence in the evidence is constrained by factors such as the number, size, or methodology quality of individual studies; lack of coherence in the chain of evidence; or limited generalizability, and the magnitude or direction of the observed effect could change when more information becomes available. Low strength of evidence indicates that the available evidence is insufficient to assess effects because of the limited number of studies, significant flaws in study design or methods indicated by low PEDro scores, inconsistency of findings across studies, or limited generalizability.

## Results

**Fig. 1:**
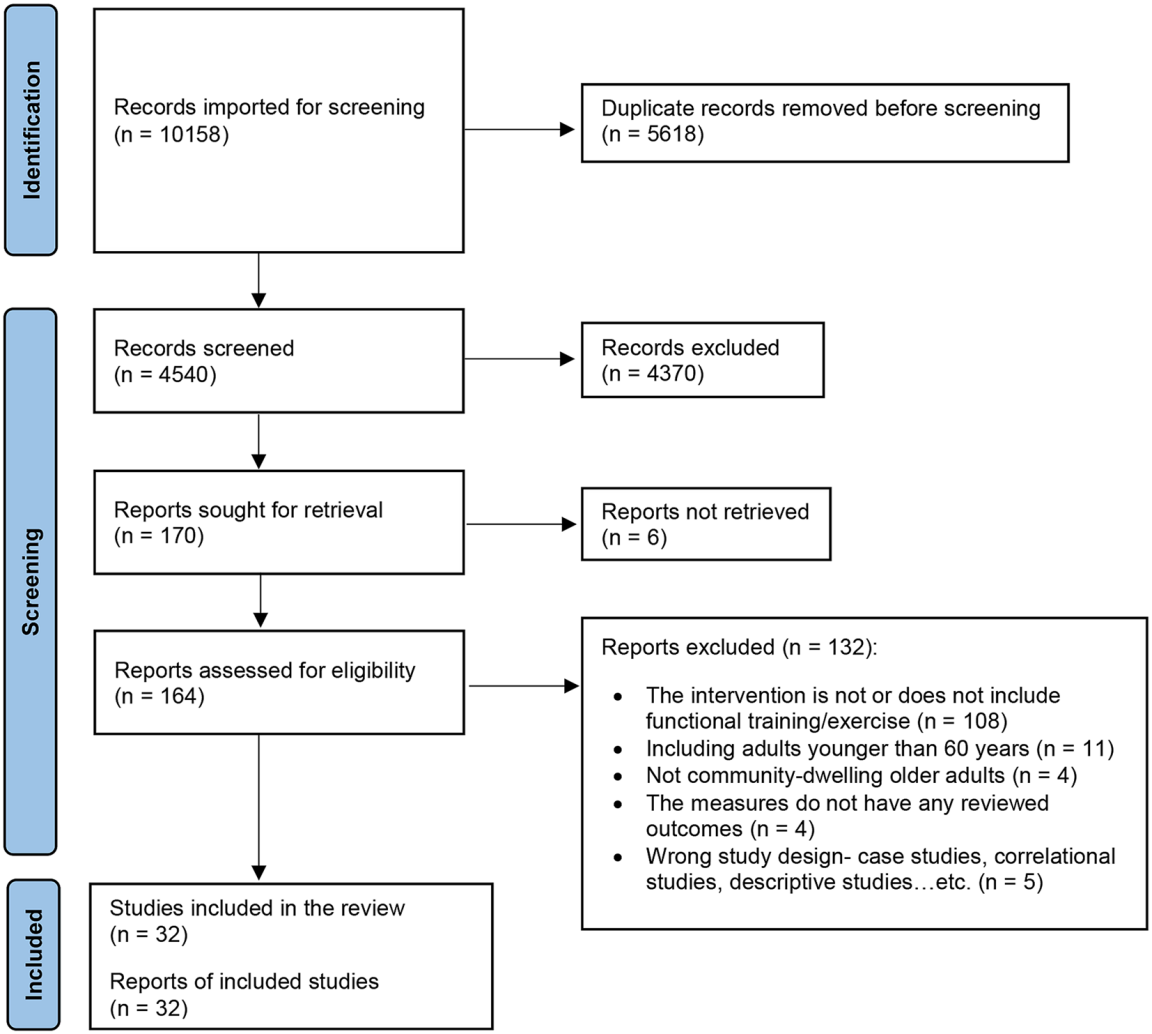
Flow chart of the systematic review process

Of the 10,158 articles identified, 32 were included in the data analysis. The flowchart in Fig. 1 shows the screening process. Appendix C summarizes the research design, study participants and intervention characteristics, outcome measures, and relevant main findings of the included articles. Table 1 highlights the study and participant characteristics.

**Table 1:** Trial characteristics of all studies

Total *n* = 32	Number of Trials
**Publication Countries by Continent**	
America	9
Asia	7
Europe	10
Oceania	6
**Publication Years**	
2010–2014	11
2015–2019	10
2020–2024	11
**Sample Size**	
< 100	22
101–300	7
301–500	3
**Types of Participants**	
General community-dwelling older adults	20
Community-dwelling older adults with cognitive impairments	9
Frail older adults	3
**Mean Age (Years)**	
70–79	20
> 80	11
Not reported	1
**% Female**	
0-49.9	3
50–100	29
**Types of Functional Training**	
Single component	22
Multi-component	8
Dual-task	2
**Duration of Intervention (Weeks)**	
< 13	22
13–24	5
25–52	5

### Characteristics of functional training

The training programs were categorized into three main categories: the single-component approach (*n* = 22), the multi-component approach (*n* = 8), and the dual-task paradigm approach (*n* = 2). The single-component approach focuses on exercise as the sole intervention, which could be functional training exercise alone or in combination with other types of physical exercises, such as resistance training. For example, the LiFE program integrates balance and muscle-strengthening exercises into everyday activities, and the 3-Step Workout for Life program combines resistance exercises with daily activity exercises [29, 30]. The multi-component approach includes other components in addition to exercise, such as combining exercises, home modifications, and caregiver support intervention with ADL training [31]. The dual-task paradigm approach requires participants to perform two tasks simultaneously, and at least one is a functional task. For example, maintaining balance while reading newspapers or carrying a tray with glasses [32].

The general intervention duration ranged from 40 to 60 min per session, with three sessions per week, and lasted 8 to 13 weeks. Healthcare professionals, such as physical or occupational therapists, typically delivered the intervention. The intervention could be home-based, center/hospital-based, or a combination of both.

### Outcome measures

Outcome measures were categorized into ADL/IADL, physical functioning, and cognitive function. See Appendix D for the list of reviewed measures. Twenty-one studies measured personal ADL and/or instrumental ADL outcomes, predominantly self-reported or observation-based instruments, such as Katz’s ADL or Lawton IADL Scale. Twenty-four studies measured physical functioning, which was categorized into overall physical function (i.e., physical performance of the upper body and lower body together), lower body function (i.e., mobility or walking), upper body function (i.e., gross and fine motor hand function), and balance (i.e., static and dynamic balance). Lower body function, especially the Timed Up and Got test, is measured more often than upper body function and overall physical function. Ten studies measured cognitive functions, which were categorized into global cognitive function and domain-specific cognitive function. Global cognitive function assesses multiple domains of cognitive function concurrently, such as the Mini Mental State Examination or Neurobehavioral Cognitive Status Examination.

### Methodology quality

Table 2 shows the PEDro scale results according to the functional training approaches and participants’ characteristics. The overall level of methodological quality of the studies included was fair, with a mean score of 4.84. Specifically, 47% of the studies achieved a score of 6 or higher, indicating good methodological quality.

**Table 2:** PEDro Scale Rating

Study ID^a^	PEDro Criteria											Score
	#1	#2	#3	#4	#5	#6	#7	#8	#9	#10	#11	
General older adults: Single-component approach												
Barcelona 2023 [44]	YES	YES	NO	YES	NO	NO	YES	YES	NO	YES	YES	6
Burton 2013 [33]	YES	YES	YES	YES	NO	NO	NO	YES	YES	YES	YES	7
Clemson 2012 [29]	YES	YES	YES	YES	NO	NO	YES	NO	YES	YES	YES	7
Hu 2023 [43]	YES	YES	NO	YES	NO	NO	NO	YES	YES	YES	YES	6
Hummer 2015 [34]	YES	NA	NA	NA	NO	NO	NO	YES	NO	NA	YES	2
Karóczi 2014 [41]	YES	NA	NA	YES	NO	NO	NO	YES	YES	YES	YES	5
King 2012 [35]	YES	YES	NO	YES	NO	NO	YES	YES	YES	YES	YES	7
Li 2018 [42]	YES	NA	NA	NA	NO	NO	NO	NO	NO	NA	YES	1
Liu 2016 [38]	YES	NA	NA	NA	NO	NO	NO	NO	NO	NA	YES	1
Liu 2017 [39]	YES	YES	NO	NO	NO	NO	YES	YES	YES	YES	YES	6
Liu 2020 [30]	YES	NA	NA	NA	NO	NO	NO	NO	NO	NA	YES	1
Mohammed 2022 [36]	YES	YES	YES	NO	NO	NO	NO	NO	NO	YES	YES	4
Siemonsma 2018 [37]	YES	YES	NO	YES	NO	NO	YES	NO	YES	YES	YES	6
Solberg 2013 [40]	YES	YES	NO	YES	NO	NO	YES	NO	NO	YES	YES	5
General older adults: Multi-component approach												
Comans 2010 [45]	YES	YES	YES	YES	NO	NO	YES	NO	YES	YES	YES	7
Oh 2021 [47]	YES	YES	NO	YES	NO	NO	NO	YES	NO	YES	YES	5
Szanton 2021 [46]	YES	YES	YES	YES	NO	NO	YES	YES	YES	YES	YES	8
Todo 2021 [31]	YES	NA	NA	NA	NO	NO	NO	YES	NO	NA	YES	2
General older adults: Dual-task paradigm approach												
Halvarsson 2011 [32]	YES	YES	YES	YES	NO	NO	YES	YES	NO	YES	YES	7
Nott 2019 [48]	YES	NA	NA	YES	NO	NO	NO	NO	NO	YES	YES	3
Older adults with mild cognitive impairment: Single-component approach												
de Freitas 2021 [49]	YES	NA	NA	NA	NO	NO	NO	YES	NO	NA	YES	2
Law 2013 [18]	YES	NA	NA	NA	NO	NO	NO	YES	YES	NA	YES	3
Law 2014 [17]	YES	YES	YES	YES	NO	NO	YES	YES	YES	YES	YES	8
Law 2018 [19]	YES	NA	NA	NA	NO	NO	NO	YES	NO	NA	YES	2
Older adults with mild cognitive impairment: Multi-component approach												
Liao 2020 [50]	YES	YES	YES	YES	NO	NO	YES	NO	NO	YES	YES	6
Older adults with dementia: Single-component approach												
Hauer 2012 [51]	YES	YES	YES	YES	YES	NO	YES	YES	YES	YES	YES	9
Pedroso 2018 [52]	YES	NA	NA	YES	NO	NO	NO	NO	NO	YES	YES	3
Older adults with mild cognitive impairment or dementia: Multi-component approach												
Harwood 2023 [54]	YES	YES	YES	YES	NO	NO	NO	NO	YES	YES	YES	6
Straubmeier 2017 [53]	YES	YES	NO	YES	NO	NO	YES	NO	YES	YES	YES	6
Frail older adults: Single-component approach												
Faria 2023 [56]	YES	YES	NO	NO	NO	NO	YES	YES	YES	NO	YES	5
Oosting 2012 [55]	YES	YES	NO	YES	NO	NO	NO	YES	YES	YES	YES	6
Frail older adults: Multi-component approach												
van Lieshout 2018 [57]	YES	YES	NO	YES	NO	NO	NO	NO	NO	YES	YES	4

Note. a- First author’s last name and publication year

Note: Item 1 (not scored)- Eligibility inclusion criteria and source; Item 2- Randomization; Item 3- Concealed allocation; Item 4- Compatibility between groups, similarity at baseline; Item 5- Blinded participants; Item 6- Blinded interventionists; Item 7- Blinded assessors; Item 8- Attrition, completeness of follow up; Item 9- Intention to treat; Item 10- Between-group comparison results; Item 11- Treatment effect and measures of variability. NA- Not applicable

### Summary of Key findings

#### General community-dwelling older adults

Twenty studies recruited older adults without specifying their cognitive or frailty status. Among these, 14 used a single-component approach, four used a multi-component approach, and two used a dual-task paradigm approach.

**Single-component approach and ADL/IADL outcomes.** Nine studies measured ADL/IADL outcomes [29, 30, 33–39]. Six studies compared the single-component approach with a control group or another active intervention, such as structured resistance or balance exercise, and five reported a significant outcome [29, 33, 36, 37, 39]. The other three studies examined the effect using a pretest-posttest design, and two from the same research group showed a significant improvement [30, 38]. In summary, there is strong strength of evidence that single-component functional training can improve or maintain ADL/IADL performance in general community-dwelling older adults.

Among the seven studies with a significant outcome, two examined the LiFE program [29, 33] and three examined the 3-Step Workout for Life program [30, 38, 39]. Another two studies applied task-specific training which consisted of exercise linked with the participant’s home environment [37] and simulated daily activity training [36].

**Single-component approach and physical functioning outcomes.** Eleven studies measured physical functioning [29, 30, 33, 35, 38–44]. Seven studies compared functional training with a control group or another active intervention [29, 33, 35, 39, 40, 43, 44], and none reported a difference except three studies that reported an improvement in balance or mobility [29, 33, 44]. Two of the three studies examined the LiFE program.

The other four studies applied a pretest-posttest design [30, 38, 41, 42]. These studies measured lower body performance, but the results were mixed. The results were also mixed in two studies that measured upper body performance [30, 38]. Three studies measured mobility outcomes, and all showed a positive effect [30, 41, 42]. Two studies measured balance, and both showed a positive effect [41, 42]. In summary, there is moderate strength of evidence for using functional training as a single-component approach to improve balance and mobility in general community-dwelling older adults. However, the effect may not be greater than other structured exercise or usual care programs.

**Multi-component approach and ADL/IADL**,** physical functioning**,** and cognitive function outcomes.** The intervention components in the four multi-component studies varied and might include structured exercise, fall prevention education, home modification recommendations, home exercise programs, motor imagery training, and/or caregiver support in addition to functional training [31, 45–47]. Two studies measured ADL/IADL outcomes [31, 45]. One was a pretest-posttest trial and showed a significant improvement [31]. The other was an RCT [45], which compared multi-component functional training with a center-based exercise program. The study did not show a superior effect of the multi-component functional training. This study also measured global cognition but did not find an effect.

Three studies measured physical functioning outcomes [45–47]. All were RCTs. One trial compared multi-component functional training to a center-based exercise program, as aforementioned [45]. However, the trial found a superior effect on hand dexterity in a center-based exercise program. The second trial compared multi-component functional training to attention controls and found a superior effect of functional training on balance but not mobility [46]. The third trial compared multi-component functional training and single-component functional training with fall prevention education [47]. The trial found that both trainings improved physical functioning outcomes, but the improvement in multi-component functional training was greater. In summary, there is low strength of evidence of multi-component functional training on ADLs/IADLs and physical functioning.

**Dual-task paradigm and physical functioning and cognitive function outcomes.** Two studies applied the dual-task paradigm approach [32, 48]. Both studies compared dual-task functional training with a control group. One study showed an improvement in gait speed [32]. The other study did not show a superior effect on mobility [48]. Additionally, one of the studies measured cognitive function but did not find an effect [48]. Overall, findings from the two studies are not sufficient to make a conclusion about the effect of dual-task paradigm functional training.

#### Community-dwelling older adults with mild cognitive impairment

Five studies included older adults with mild cognitive impairment. Four of these studies used a single-component approach. One study used a multi-component approach.

**Single-component approach and ADL/IADL and cognitive function outcomes**. Four studies examined the same single-component approach, called Simulated Functional Tasks Exercise [17–19, 49]. The training program involved sorting cups and bowls while following specific rules and specific movement patterns to stimulate working memory and executive function. All studies showed an improvement in the ADL/IADL outcomes, including three pretest-posttest studies [18, 19, 49] and one RCT, which compared the program to a computer-based cognitive training program [17]. Furthermore, improvements were detected in overall cognitive function, memory, problem-solving, and executive function. Single-component functional training seems beneficial for improving ADL/IADL performance and cognitive outcomes in older adults with mild cognitive impairment. However, the strength of the evidence was regarded as moderate due to certain limitations. All four studies were conducted by the same research group and investigated the same functional training program. Furthermore, three of these studies applied a single-group pretest-posttest research design.

**Multi-component approach and ADL/IADL and cognitive function outcomes.** Only one study applied a multi-component approach [50]. The approach involved VR-based physical and cognitive training using everyday tasks, such as window cleaning and food preparation. Compared with combined multimodal exercise and cognitive training, the intervention had positive effects on IADLs but not on global cognition, memory, or executive function.

#### Community-dwelling older adults with dementia

Two studies recruited older adults with dementia and used a single-component approach. Additionally, two other studies recruited older adults with dementia or mild cognitive impairment and used a multi-component approach.

**Single-component approach and ADL/IADL and physical functioning outcomes.** Function training in one study focused on functionally relevant muscle groups and ADL-related motor functions, such as stair climbing [51]. This intervention was more effective in improving lower body function, mobility, and balance than low-intensity multimodal exercise in older adults with dementia. Another study applied simulated locomotion and ADLs to people with dementia [52]. There were no significant differences in ADLs, balance, overall physical function, or global cognitive outcomes when compared to a social gathering group.

**Multi-component approach and ADL/IADL**,** physical functioning**,** and cognitive function outcomes**. One study combined sensorimotor exercise, cognitive training, and ADLs as activations [53]. Compared to usual care in daycare centers, the multi-component approach group significantly maintained ADL function and global cognition. The other study combined physical exercise, daily activities, community participation, risk enablement (positive risk taking), and environmental assessment [54]. Although the multi-component intervention group did not outperform a simple fall prevention group on the outcomes of ADLs/IADLs, balance, and mobility, the intervention yielded better outcomes in executive function and visual-spatial working memory. In short, the strength of evidence for multi-component functional training for people with dementia is low because of limited studies and mixed results.

### Community-dwelling older adults with frailty

Three studies recruited older adults using frailty-specific screening criteria and all were RCTs. Two studies used a single-component approach, and one used a multi-component approach.

**Single-component approach and ADL/IADL and physical functioning outcomes**. Both studies applied multi-modal exercise. One combined tailored walking with activity exercises [55]. The study showed a superior effect on one of the physical functioning tests of the lower extremities (i.e., 6-minute walk) when compared to an educational control group. The other combined strength training, endurance, balance, and flexibility exercises with ADL training [56]. The group improved in ADLs and all lower extremity physical functioning measures but not in IADLs after program completion. There was low strength of evidence that single-component functional training can improve the physical functioning of the lower extremities for frail older adults.

**Multi-component approach and ADL/IADL and physical functioning outcomes**. The study combined medication use and safety and nutritional status optimization with exercise and ADL training, such as walking outdoors and shopping [57]. Compared to a control group, the intervention did not have a superior effect on the ADLs or the physical functional outcomes.

## Discussion

This systematic review examined the effects of functional training in community-dwelling older adults. The findings suggest a strong effect of single-component functional training on improving or maintaining ADL/IADL performance in older adults without cognitive impairment and frailty. However, the effect on physical functioning, such as balance and mobility, was not superior to other exercise programs. Consistent effects of the Simulated Functional Tasks Exercise program as a single-component approach on the ADLs/IADLs outcomes and cognitive functions were identified in older adults with mild cognitive impairment. When functional training was combined with other intervention components using a multi-component approach, the effects varied greatly and yielded a low level of evidence. The review also identified functional training that applied the dual-task paradigm, but the number of studies is limited.

Earlier research highlighted the positive effects of functional training on balance, mobility, and ADL/IADL for community-dwelling older adults [14]. The findings of the current review extended these findings, demonstrating that single-component functional training has a superior effect on ADL/IADL outcomes compared to other active interventions. Note that only a few single-component functioning training programs combined functioning with other exercises, such as aerobic exercise, in this review. However, the superiority of single-component functional training in improving balance and mobility over other structured exercise programs is limited, suggesting that both functional training and structured exercise are equally effective in enhancing physical functioning for older adults. This could be that muscle strengthening was blended in most single-component functioning training programs, such as LiFE and 3-Step Workout for Life. These findings affirm the significance of incorporating functional training for older adults to simultaneously preserve their physical and functional performance, which is crucial for maintaining independent living.

The majority of functional training programs included in the review are motor-centric. For the training to be considered “functional” in this review, the training must incorporate movements that complete daily tasks more than simple ambulatory actions, such as walking. Functional training goes beyond traditional structured physical fitness exercises because it applies the principle of specificity [15]—what is specifically trained is improved—to induce neuromuscular adaptations to meet the versatile demands of daily tasks. When functional training is applied as a single-component approach, regardless of whether it is combined with other types of exercise or not, the effect on the ADL/IADL outcomes for general community-dwelling older adults is robust.

In contrast, the effect of functional training combined with other interventions, such as education or home modification, delivered as a multi-component approach for general community-dwelling older adults, is limited. Only four studies included in the review applied a multi-component approach in general community-dwelling older adults. Notably, three of these studies were fall prevention trials, and only one included the ADL/IADL measure. Functional training in these trials utilizes daily activities that challenge older adults’ balance and muscle strength of the lower extremities. The application of a multi-component approach to reduce older adults’ risk of falls is well established [58]. The multi-component approach may help older adults to perform ADLs/IADLs safer.

Performing everyday tasks necessitates both motor and cognitive abilities. The cognitive demand in functional training was increased in several reviewed trials, particularly those involving older adults with cognitive impairment. The cognitive demand can be increased through the dual-task paradigm [59], which simultaneously challenges both motor and cognitive abilities, and can be categorized into motor-cognitive or motor-motor. The functional training paradigm can be motor-cognitive; for example, older adults perform one functional task, such as climbing stairs, while simultaneously counting even numbers. The paradigm can also be motor-motor; for example, older adults perform two functional tasks, such as carrying a tray with glasses of water while walking around cones. However, only two trials in this review applied the dual-task paradigm, and none measured the outcomes of ADLs/IADLs [32, 48]. Additionally, the effects on physical functioning and cognitive function are inconclusive due to the limited number of studies.

The present review identified four studies of Simulated Functional Tasks Exercise, a single-component approach, in older adults with mild cognitive impairment [17–19, 49]. This particular exercise may be considered applying the motor-motor dual-task paradigm [59]. For example, the exercise manipulates the sequence of steps (forward or backward), requires bimanual coordination and body midline crossing movements, and uses task switching and interference to challenge working memory and executive function. Additionally, the training effects on memory, executive function, and problem-solving are greater compared to computer-based cognitive training. These findings support the principle of specificity [15] and the compound effect of combined cognitive and physical exercise through functional training. Although promising, the consistency of findings across trials warrants a cautious interpretation because these trials were conducted by the same research group, and only one was an RCT.

Virtual reality enables individuals to immerse themselves in a simulated, three-dimensional environment, allowing them to interact with the virtual space as if they were physically present through specialized devices. Virtual reality-based exergaming intervention, which combines exercise and gaming, has been shown to improve global cognitive function, memory, and executive functions in people with mild cognitive impairment [60]. Although the current review did not include virtual reality as a search term, the review identified one trial using virtual reality to replicate physically demanded tasks (e.g., window cleaning) or cognitively demanded tasks (e.g., use of public transportation) [50]. The study showed specificity effects (superior outcomes in IADLs) rather than compound effects (superior cognitive function outcomes) compared to concurrent multimodal exercise and cognitive training in people with mild cognitive impairment. While virtual reality allows people to perform functional tasks without the physical setup, whether they exert the same cognitive and physical effort in virtual reality as they would in the real world and yield a compound effect remains an open question. The study indicates that virtual reality is a feasible platform to deliver functional training.

Three trials tested the effects of functional training in older adults with mild to moderate dementia using either a single-component approach [51, 52] or a multi-component approach [53]. Notably, functional training in all three trials was motor-centric and was compared to an active control group who received low-intensity exercise or social and recreational activities. The limited number of trials and the use of a comparative-effectiveness research design could explain the mixed findings of functional training for older adults with dementia. In addition, the cognitive stimuli offered by recreational activities in the comparison group could be more cognitively enriching and engaging than motor-centric functional training. Maintaining an active lifestyle is assumed to offer multiple health benefits [61]. Although no definitive effects were identified for those with dementia in this review, these trials demonstrate that functional training may be offered as an alternative for encouraging physical activity in people with dementia.

Frailty increases older adults’ vulnerability to daily stressors and the risk of adverse health outcomes due to cumulative deteriorations in various physiological systems [62]. Physical exercise is essential for managing frailty [63]. However, only three trials examined functional training in frail older adults, presenting mixed results on physical functioning. Incorporating functional training to improve physical functioning and ADL performance in frail older adults requires additional research.

### Limitations

While the current review aims to evaluate the effects of functional training, the training effects cannot be isolated in studies that incorporate other types of exercise or components in the intervention program. Researchers in these studies often aimed to address complex issues, such as falls or frailty, making a complex intervention design appropriate. Additionally, some studies in the category of general community-dwelling older adults might have included frail older adults, especially those recruited participants from assisted living [34, 44]. If these studies were recognized, the strength of evidence of functional training in frail older adults would remain low. Finally, a meta-analysis was not conducted because of the heterogeneity in functional training and outcome measures across studies.

## Conclusion

The current review expands upon previous research in several key areas. Specifically, this review examines multi-component and dual training paradigms in addition to the single-component approach. Furthermore, it includes data from older adults with cognitive impairment or frailty and incorporates cognitive function as an outcome measure. Functional training as a single-component is recommended to prevent late-life ADL disability in general community-dwelling older adults. The training may also improve cognitive function in people with mild cognitive impairment. Although the evidence in people with dementia or frailty is limited, these trials represent an important step toward expanding functional training to populations highly susceptible to functional decline.

## Electronic supplementary material

Below is the link to the electronic supplementary material.

Supplementary Material 1

Supplementary Material 2

Supplementary Material 3

Supplementary Material 4

## Data Availability

All data generated or analyzed during this study are included in this published article [and its supplementary information files].

## Abbreviations

ADLs: Activities of daily living
IADLs: Instrumental activities of daily living
