# Correction: Is functional training functional? a systematic review of its effects in community-dwelling older adults

**DOI:** 10.1186/s11556-025-00369-8

**Published:** 2025-02-11

**Authors:** Chiung-ju Liu, Wen-Pin Chang, Yun Chan Shin, Yi-Ling Hu, Jane Morgan-Daniel

**Affiliations:** 1 Department of Occupational Therapy, College of Public Health and Health Professions, University of Florida, 1225 Center Drive, P.O. Box 100164, Gainesville, FL 32610-0164 USA; 2 Department of Occupational Therapy, College of Health Professions, University of Texas Rio Grande Valley, Edinburg, TX USA; 3 Department of Occupational Therapy, Chang Gung University, Taoyuan, Taiwan; 4 Health Science Center Libraries, University of Florida, Gainesville, USA

**Correction:** ***Eur Rev Aging Phys Act*** **21, 32 (2024)**

10.1186/s11556-024-00366-3

Following the publication of the original article [1], the city of academic affiliation for Wen-Pin Chang was incorrectly given as ‘Gainesville’ but should have been ‘Edinburg’.

This has been corrected above and the original article [1] has been updated.
