# Supplementary material for: Is functional training functional? a systematic review of its effects in community-dwelling older adults
Source: Eur Rev Aging Phys Act. 2024 Dec 21;21:32. doi: 10.1186/s11556-024-00366-3 (PMC11664925; doi:10.1186/s11556-024-00366-3)
Supplement: Supplementary file 2 — Supplementary Material 2 [file 11556_2024_366_MOESM1_ESM.docx]

**Appendix B: Search Terms and Strategies**

**CINAHL**

- Population: Older adults (65 years and over), age-related change only (no other conditions), community dwelling

Terms for older adults

Title/Abstract: (“older adult*” OR “old adult*” OR elder* OR “old people” OR “older people” OR “old person*” OR “older person*” OR “older communit*” OR "older population*” OR "senior people" OR "senior person*" OR “senior citizen*” OR "senior communit*” OR "senior population*” OR geriatric* OR frail* OR prefrail* OR “pre-frail*” OR “oldest old” OR “old age” OR “old aged” OR “older age” OR “older aged” OR “age related cognitive impairment*” OR “age-related cognitive impairment*” OR “ARCI” OR fragile OR fragility OR “aging adult*” OR “ageing adult*”) OR (MH "Aged") OR (MH "Aged, 80 and Over") OR (MH "Centenarians") OR (MH "Frail Elderly") OR (MH "Rehabilitation, Geriatric"))

AND

Terms for community dwelling

TIAB (“independent living” OR “living independently” OR “live independently” OR “lives independently” OR “community dwelling*” OR “community-dwelling*” OR “retirement communit*” OR “retirement cent*” OR “retirement life care cent*” OR “age in place” OR “aging in place” OR “ageing in place” OR “ages in place” OR “aged in place” OR “age at home” OR “aging at home” OR “ageing at home” OR “ages at home” OR “aged at home” OR “private residenc*” OR “domestic environment*” OR “home environment*” OR “reside at home” OR “resides at home” OR “residing at home” OR “resided at home” OR “assisted living” OR “living at home” OR “lives at home” OR “live at home” OR “lived at home” OR “community living” OR “congregate hous*”) OR (MH "Community Living") OR (MH "Assisted Living") OR (MH "Housing for the Elderly"))

AND

Terms for functional training

TIAB (“functional task*” OR “functional exercis*” OR “functional therap*” OR “functional skill*” OR “functional performance” OR “functional activit*” OR “functional occupation*” OR “functional train*” OR “functional retrain*” OR “functional rehab*” OR “functional intervention*” OR “functional strength task*” OR “functional strength exercise*” OR “functional strength therap*” OR “functional strength skill*” OR “functional strength perform*” OR “functional strength activit*” OR “functional strength occupation*” OR “functional strength train*” OR “functional strength retrain*” OR “functional strength rehab*” OR “functional strength intervention*” OR “functional oriented rehab” OR “functional-oriented rehab*” OR (functional AND specificity) OR “occupational task*” OR “occupational exercis*” OR “occupational skill*” OR “occupational performance” OR “occupational activit*” OR “occupational train*” OR “occupational retrain*” OR “occupational rehab*” OR “occupational intervention*” OR “occupational coordination” OR “occupational co-ordination” OR (occupational AND specificity) OR “chronic activity limitation*” OR “chronic limitation of activit*” OR “daily life task*” OR “daily life exercis*” OR “daily life therap*” OR “daily life skill*” OR “daily life performance” OR “daily life activit*” OR “daily life occupational” OR “daily life train*” OR “daily life retrain*” OR “daily life rehab*” OR “daily life intervention*” OR “daily life coordination” OR “daily life co-ordination” OR “daily lives task*” OR “daily lives exercis*” OR “daily lives therap*” OR “daily lives skill*” OR “daily lives performance” OR “daily lives activit*” OR “daily lives occupational” OR “daily lives train*” OR “daily lives retrain*” OR “daily lives rehab*” OR “daily lives intervention*” OR “daily lives coordination*” OR “daily lives co-ordination” OR “psychomotor task*” OR “psychomotor exercis*” OR “psychomotor therap*” OR “psychomotor skill*” OR “psychomotor performance” OR “psychomotor activit*” OR “psychomotor occupation*” OR “psychomotor train*” OR “psychomotor retrain*” OR “psychomotor rehab*” OR “psychomotor intervention*” OR (psychomotor AND specificity) OR “life integrat*” OR “lifestyle integrat*” OR “daily living task*” OR “daily living exercis*” OR “daily living therap*” OR “daily living skill*” OR “daily living performance” OR “daily living activit*” OR “daily living occupational” OR “daily living train*” OR “daily living retrain*” OR “daily living rehab*” OR “daily living intervention*” OR “daily living coordination” OR “daily living co-ordination” OR “daily task*” OR “daily activity task*” OR “daily activities task*” “daily activity performance” OR “daily activities performance” OR “daily activity occupation*” OR “daily activities occupation*” OR “daily activity train*” OR “daily activities train*” OR “daily activity retrain*” OR “daily activities retrain*” OR “daily activity rehab*” OR “daily activities rehab*” OR “daily activity intervention*” OR “daily activities intervention*” OR “daily activity coordination” OR “daily activity co-ordination” OR “daily activities coordination” OR “daily activities co-ordination” OR “ADAP” OR “everyday task*” OR “every day task*” OR “everyday activit*” OR “every day activit*” OR “every day occupational” OR “everyday occupational” OR “everyday living task*” OR “every day living task*” OR “everyday living exercis*” OR “every day living exercis*” OR “everyday living therap*” OR “every day living therap*” OR “everyday living skill*” OR “every day living skill*” OR “everyday living performance” OR “every day living performance” OR “every day living activit*” OR “everyday living activit*” OR “everyday living occupation*” OR “every day living occupation*” OR “everyday living train*” OR “every day living train*” OR “every day living retrain*” OR “everyday living train*” OR “every day living rehab*” OR “everyday living rehab*” OR “every day living intervention*” OR “everyday living intervention*” OR “every day living coordination” OR “everyday living coordination” OR “every day living co-ordination” OR “everyday living co-ordination” OR “every day life task*” OR “everyday life task*” OR “everyday life exercis*” OR “every day life exercis*” OR “every day life therap*” OR “everyday life therap*” OR “everyday life skill*” OR “every day life skill*” OR “every day life performance” OR “everyday life performance” OR “everyday life activit*” OR “every day life activit*” OR “every day life occupation*” OR “everyday life occupation*” OR “every day life train*” OR “everyday life train*” OR “every day life retrain*” OR “everyday life retrain*” OR “every day life rehab*” OR “everyday life rehab*” OR “everyday life intervention*” OR “every day life intervention*” OR “every day life coordination” OR “everyday life coordination” OR “every day life co-ordination” OR “everyday life co-ordination” OR “self care task*” OR “self-care task*” OR “self care exercis*” OR “self-care exercis*” OR “self care therap*” OR “self-care therap*” OR “self care skill*” OR “self-care skill*” OR “self care performance” OR “self-care performance” OR “self care activit*” OR “self-care activit*” OR “self care occupation*” OR “self-care occupation*” OR “self care train*” OR “self-care train*” OR “self care retrain*” OR “self-care retrain*” OR “self care rehab*” OR “self-care rehab*” OR “self care intervention*” OR “self-care intervention*”) OR (MH "Functional Training")

OR

(shower* OR bathe OR bathes OR bathing OR “bathroom transfer*” OR “personal hygiene” OR “toilet hygiene” OR grooming OR dressing OR “getting dressed” OR laundry OR vacuuming OR hoovering OR ironing OR housekeep* OR “household management” OR “housework*” OR “managing house*” OR “manage house*” OR “managed house*” OR homemak* OR cleaning OR “purposeful movement*” OR “purposeful activit*” OR ADL OR ADLS OR IADL OR IADLS OR “activity of daily living” OR “activities of daily living”) OR (MH "Activities of Daily Living") OR (MH "Bathing and Baths") OR (MH "Grooming") OR (MH "Dressing")) AND (task* OR exercis* OR performance OR train* OR retrain*OR rehab* OR intervention* OR specificity)

NOT

TI: (protocol* OR conference* OR proceedings OR “book review” OR editorial)

AND

Limits: 2010-2021; English; NOT Magazines OR CEUs

**Web of Science**

- Population: Older adults (65 years and over), age-related change only (no other conditions), community dwelling

Terms for older adults

Topic: (“older adult*” OR “old adult*” OR elder* OR “old people” OR “older people” OR “old person*” OR “older person*” OR “older communit*” OR "older population*” OR "senior people" OR "senior person*" OR “senior citizen*” OR "senior communit*” OR "senior population*” OR geriatric* OR frail* OR prefrail* OR “pre-frail*” OR “oldest old” OR “old age” OR “old aged” OR “older age” OR “older aged” OR “age related cognitive impairment*” OR “age-related cognitive impairment*” OR “ARCI” OR fragile OR fragility OR “aging adult*” OR “ageing adult*”)

AND

Terms for community dwelling

Topic (“independent living” OR “living independently” OR “live independently” OR “lives independently” OR “community dwelling*” OR “community-dwelling*” OR “retirement communit*” OR “retirement cent*” OR “retirement life care cent*” OR “age in place” OR “aging in place” OR “ageing in place” OR “ages in place” OR “aged in place” OR “age at home” OR “aging at home” OR “ageing at home” OR “ages at home” OR “aged at home” OR “private residenc*” OR “domestic environment*” OR “home environment*” OR “reside at home” OR “resides at home” OR “residing at home” OR “resided at home” OR “assisted living” OR “living at home” OR “lives at home” OR “live at home” OR “lived at home” OR “community living” OR “congregate hous*”)

AND

Terms for functional training

Topic (“functional task*” OR “functional exercis*” OR “functional therap*” OR “functional skill*” OR “functional performance” OR “functional activit*” OR “functional occupation*” OR “functional train*” OR “functional retrain*” OR “functional rehab*” OR “functional intervention*” OR “functional strength task*” OR “functional strength exercise*” OR “functional strength therap*” OR “functional strength skill*” OR “functional strength perform*” OR “functional strength activit*” OR “functional strength occupation*” OR “functional strength train*” OR “functional strength retrain*” OR “functional strength rehab*” OR “functional strength intervention*” OR “functional oriented rehab” OR “functional-oriented rehab*” OR (functional AND specificity) OR “occupational task*” OR “occupational exercis*” OR “occupational skill*” OR “occupational performance” OR “occupational activit*” OR “occupational train*” OR “occupational retrain*” OR “occupational rehab*” OR “occupational intervention*” OR “occupational coordination” OR “occupational co-ordination” OR (occupational AND specificity) OR “chronic activity limitation*” OR “chronic limitation of activit*” OR “daily life task*” OR “daily life exercis*” OR “daily life therap*” OR “daily life skill*” OR “daily life performance” OR “daily life activit*” OR “daily life occupational” OR “daily life train*” OR “daily life retrain*” OR “daily life rehab*” OR “daily life intervention*” OR “daily life coordination” OR “daily life co-ordination” OR “daily lives task*” OR “daily lives exercis*” OR “daily lives therap*” OR “daily lives skill*” OR “daily lives performance” OR “daily lives activit*” OR “daily lives occupational” OR “daily lives train*” OR “daily lives retrain*” OR “daily lives rehab*” OR “daily lives intervention*” OR “daily lives coordination*” OR “daily lives co-ordination” OR “psychomotor task*” OR “psychomotor exercis*” OR “psychomotor therap*” OR “psychomotor skill*” OR “psychomotor performance” OR “psychomotor activit*” OR “psychomotor occupation*” OR “psychomotor train*” OR “psychomotor retrain*” OR “psychomotor rehab*” OR “psychomotor intervention*” OR (psychomotor AND specificity) OR “life integrat*” OR “lifestyle integrat*” OR “daily living task*” OR “daily living exercis*” OR “daily living therap*” OR “daily living skill*” OR “daily living performance” OR “daily living activit*” OR “daily living occupational” OR “daily living train*” OR “daily living retrain*” OR “daily living rehab*” OR “daily living intervention*” OR “daily living coordination” OR “daily living co-ordination” OR “daily task*” OR “daily activity task*” OR “daily activities task*” “daily activity performance” OR “daily activities performance” OR “daily activity occupation*” OR “daily activities occupation*” OR “daily activity train*” OR “daily activities train*” OR “daily activity retrain*” OR “daily activities retrain*” OR “daily activity rehab*” OR “daily activities rehab*” OR “daily activity intervention*” OR “daily activities intervention*” OR “daily activity coordination” OR “daily activity co-ordination” OR “daily activities coordination” OR “daily activities co-ordination” OR “ADAP” OR “everyday task*” OR “every day task*” OR “everyday activit*” OR “every day activit*” OR “every day occupational” OR “everyday occupational” OR “everyday living task*” OR “every day living task*” OR “everyday living exercis*” OR “every day living exercis*” OR “everyday living therap*” OR “every day living therap*” OR “everyday living skill*” OR “every day living skill*” OR “everyday living performance” OR “every day living performance” OR “every day living activit*” OR “everyday living activit*” OR “everyday living occupation*” OR “every day living occupation*” OR “everyday living train*” OR “every day living train*” OR “every day living retrain*” OR “everyday living train*” OR “every day living rehab*” OR “everyday living rehab*” OR “every day living intervention*” OR “everyday living intervention*” OR “every day living coordination” OR “everyday living coordination” OR “every day living co-ordination” OR “everyday living co-ordination” OR “every day life task*” OR “everyday life task*” OR “everyday life exercis*” OR “every day life exercis*” OR “every day life therap*” OR “everyday life therap*” OR “everyday life skill*” OR “every day life skill*” OR “every day life performance” OR “everyday life performance” OR “everyday life activit*” OR “every day life activit*” OR “every day life occupation*” OR “everyday life occupation*” OR “every day life train*” OR “everyday life train*” OR “every day life retrain*” OR “everyday life retrain*” OR “every day life rehab*” OR “everyday life rehab*” OR “everyday life intervention*” OR “every day life intervention*” OR “every day life coordination” OR “everyday life coordination” OR “every day life co-ordination” OR “everyday life co-ordination” OR “self care task*” OR “self-care task*” OR “self care exercis*” OR “self-care exercis*” OR “self care therap*” OR “self-care therap*” OR “self care skill*” OR “self-care skill*” OR “self care performance” OR “self-care performance” OR “self care activit*” OR “self-care activit*” OR “self care occupation*” OR “self-care occupation*” OR “self care train*” OR “self-care train*” OR “self care retrain*” OR “self-care retrain*” OR “self care rehab*” OR “self-care rehab*” OR “self care intervention*” OR “self-care intervention*”)

OR

(shower* OR bathe OR bathes OR bathing OR “bathroom transfer*” OR “personal hygiene” OR “toilet hygiene” OR grooming OR dressing OR “getting dressed” OR laundry OR vacuuming OR hoovering OR ironing OR housekeep* OR “household management” OR “housework*” OR “managing house*” OR “manage house*” OR “managed house*” OR homemak* OR cleaning OR “purposeful movement*” OR “purposeful activit*” OR ADL OR ADLS OR IADL OR IADLS OR “activity of daily living” OR “activities of daily living”) AND (task* OR exercis* OR performance OR train* OR retrain* OR rehab* OR intervention* OR specificity)

NOT

(TI: protocol* OR conference* OR proceedings OR “book review” OR editorial)

AND limits

English (Languages) and 2010 or 2011 or 2012 or 2013 or 2014 or 2015 or 2016 or 2017 or 2018 or 2021 or 2020 or 2019 (Publication Years) and Articles or Review Articles or Early Access (Document Types) and Engineering or Computer Science or Science Technology Other Topics or Environmental Sciences Ecology or Chemistry or Telecommunications or Physics or Business Economics or Mathematical Computational Biology or Robotics or Architecture or Automation Control Systems or Linguistics or Materials Science or Public Administration or Radiology Nuclear Medicine Medical Imaging or Veterinary Sciences (Exclude – Research Areas)

**AGELINE**

- Population: Older adults (65 years and over), age-related change only (no other conditions), community dwelling

Terms for older adults

Title/Abstract: (“older adult*” OR “old adult*” OR elder* OR “old people” OR “older people” OR “old person*” OR “older person*” OR “older communit*” OR "older population*” OR "senior people" OR "senior person*" OR “senior citizen*” OR "senior communit*” OR "senior population*” OR geriatric* OR frail* OR prefrail* OR “pre-frail*” OR “oldest old” OR “old age” OR “old aged” OR “older age” OR “older aged” OR “age related cognitive impairment*” OR “age-related cognitive impairment*” OR “ARCI” OR fragile OR fragility OR “aging adult*” OR “ageing adult*”) OR (DE "75 " OR DE "80 " OR DE "85 " OR DE "90 " OR DE "95 " OR DE "Centenarians" OR DE "Old Old" OR DE "Young Old" OR DE "60 " OR DE "65 " OR DE "70 " OR DE "Older Adults" OR DE "Frail Elderly")

AND

Terms for community dwelling

TIAB (“independent living” OR “living independently” OR “live independently” OR “lives independently” OR “community dwelling*” OR “community-dwelling*” OR “retirement communit*” OR “retirement cent*” OR “retirement life care cent*” OR “age in place” OR “aging in place” OR “ageing in place” OR “ages in place” OR “aged in place” OR “age at home” OR “aging at home” OR “ageing at home” OR “ages at home” OR “aged at home” OR “private residenc*” OR “domestic environment*” OR “home environment*” OR “reside at home” OR “resides at home” OR “residing at home” OR “resided at home” OR “assisted living” OR “living at home” OR “lives at home” OR “live at home” OR “lived at home” OR “community living” OR “congregate hous*”) OR (DE "Independent Living" OR DE "Noninstitutionalized Populations" OR DE "Continuing Care Retirement Communities" OR DE "Retirement Communities" OR DE "Naturally Occurring Retirement Communities" OR DE "Aging in Place" OR DE "Assisted Living Facilities" OR DE "Congregate Housing" OR DE "Retirement Housing")

AND

Terms for functional training

TIAB (“functional task*” OR “functional exercis*” OR “functional therap*” OR “functional skill*” OR “functional performance” OR “functional activit*” OR “functional occupation*” OR “functional train*” OR “functional retrain*” OR “functional rehab*” OR “functional intervention*” OR “functional strength task*” OR “functional strength exercise*” OR “functional strength therap*” OR “functional strength skill*” OR “functional strength perform*” OR “functional strength activit*” OR “functional strength occupation*” OR “functional strength train*” OR “functional strength retrain*” OR “functional strength rehab*” OR “functional strength intervention*” OR “functional oriented rehab” OR “functional-oriented rehab*” OR (functional AND specificity) OR “occupational task*” OR “occupational exercis*” OR “occupational skill*” OR “occupational performance” OR “occupational activit*” OR “occupational train*” OR “occupational retrain*” OR “occupational rehab*” OR “occupational intervention*” OR “occupational coordination” OR “occupational co-ordination” OR (occupational AND specificity) OR “chronic activity limitation*” OR “chronic limitation of activit*” OR “daily life task*” OR “daily life exercis*” OR “daily life therap*” OR “daily life skill*” OR “daily life performance” OR “daily life activit*” OR “daily life occupational” OR “daily life train*” OR “daily life retrain*” OR “daily life rehab*” OR “daily life intervention*” OR “daily life coordination” OR “daily life co-ordination” OR “daily lives task*” OR “daily lives exercis*” OR “daily lives therap*” OR “daily lives skill*” OR “daily lives performance” OR “daily lives activit*” OR “daily lives occupational” OR “daily lives train*” OR “daily lives retrain*” OR “daily lives rehab*” OR “daily lives intervention*” OR “daily lives coordination*” OR “daily lives co-ordination” OR “psychomotor task*” OR “psychomotor exercis*” OR “psychomotor therap*” OR “psychomotor skill*” OR “psychomotor performance” OR “psychomotor activit*” OR “psychomotor occupation*” OR “psychomotor train*” OR “psychomotor retrain*” OR “psychomotor rehab*” OR “psychomotor intervention*” OR (psychomotor AND specificity) OR “life integrat*” OR “lifestyle integrat*” OR “daily living task*” OR “daily living exercis*” OR “daily living therap*” OR “daily living skill*” OR “daily living performance” OR “daily living activit*” OR “daily living occupational” OR “daily living train*” OR “daily living retrain*” OR “daily living rehab*” OR “daily living intervention*” OR “daily living coordination” OR “daily living co-ordination” OR “daily task*” OR “daily activity task*” OR “daily activities task*” “daily activity performance” OR “daily activities performance” OR “daily activity occupation*” OR “daily activities occupation*” OR “daily activity train*” OR “daily activities train*” OR “daily activity retrain*” OR “daily activities retrain*” OR “daily activity rehab*” OR “daily activities rehab*” OR “daily activity intervention*” OR “daily activities intervention*” OR “daily activity coordination” OR “daily activity co-ordination” OR “daily activities coordination” OR “daily activities co-ordination” OR “ADAP” OR “everyday task*” OR “every day task*” OR “everyday activit*” OR “every day activit*” OR “every day occupational” OR “everyday occupational” OR “everyday living task*” OR “every day living task*” OR “everyday living exercis*” OR “every day living exercis*” OR “everyday living therap*” OR “every day living therap*” OR “everyday living skill*” OR “every day living skill*” OR “everyday living performance” OR “every day living performance” OR “every day living activit*” OR “everyday living activit*” OR “everyday living occupation*” OR “every day living occupation*” OR “everyday living train*” OR “every day living train*” OR “every day living retrain*” OR “everyday living train*” OR “every day living rehab*” OR “everyday living rehab*” OR “every day living intervention*” OR “everyday living intervention*” OR “every day living coordination” OR “everyday living coordination” OR “every day living co-ordination” OR “everyday living co-ordination” OR “every day life task*” OR “everyday life task*” OR “everyday life exercis*” OR “every day life exercis*” OR “every day life therap*” OR “everyday life therap*” OR “everyday life skill*” OR “every day life skill*” OR “every day life performance” OR “everyday life performance” OR “everyday life activit*” OR “every day life activit*” OR “every day life occupation*” OR “everyday life occupation*” OR “every day life train*” OR “everyday life train*” OR “every day life retrain*” OR “everyday life retrain*” OR “every day life rehab*” OR “everyday life rehab*” OR “everyday life intervention*” OR “every day life intervention*” OR “every day life coordination” OR “everyday life coordination” OR “every day life co-ordination” OR “everyday life co-ordination” OR “self care task*” OR “self-care task*” OR “self care exercis*” OR “self-care exercis*” OR “self care therap*” OR “self-care therap*” OR “self care skill*” OR “self-care skill*” OR “self care performance” OR “self-care performance” OR “self care activit*” OR “self-care activit*” OR “self care occupation*” OR “self-care occupation*” OR “self care train*” OR “self-care train*” OR “self care retrain*” OR “self-care retrain*” OR “self care rehab*” OR “self-care rehab*” OR “self care intervention*” OR “self-care intervention*”)

OR

(shower* OR bathe OR bathes OR bathing OR “bathroom transfer*” OR “personal hygiene” OR “toilet hygiene” OR grooming OR dressing OR “getting dressed” OR laundry OR vacuuming OR hoovering OR ironing OR housekeep* OR “household management” OR “housework*” OR “managing house*” OR “manage house*” OR “managed house*” OR homemak* OR cleaning OR “purposeful movement*” OR “purposeful activit*” OR ADL OR ADLS OR IADL OR IADLS OR “activity of daily living” OR “activities of daily living”) OR (DE "Instrumental Activities of Daily Living" OR DE "Bathing" OR DE "Personal Care" OR DE "Activities of Daily Living") AND (task* OR exercis* OR performance OR train* OR retrain* OR rehab* OR intervention* OR specificity)

NOT

TI: (protocol* OR conference* OR proceedings OR “book review” OR editorial)

AND

Limits: 2010-2021 (all English and Academic Journals)

**PubMed**

Terms for older adults

Title/Abstract: (“older adult*” OR “old adult*” OR elder* OR “old people” OR “older people” OR “old person*” OR “older person*” OR “older communit*” OR "older population*” OR "senior people" OR "senior person*" OR “senior citizen*” OR "senior communit*” OR "senior population*” OR geriatric* OR frail* OR prefrail* OR “pre-frail*” OR “oldest old” OR “old age” OR “old aged” OR “older age” OR “older aged” OR “age related cognitive impairment*” OR “age-related cognitive impairment*” OR “ARCI” OR fragile OR fragility OR “aging adult*” OR “ageing adult*”) OR ("Aged"[Mesh])

AND

Terms for community dwelling

TIAB (“independent living” OR “living independently” OR “live independently” OR “lives independently” OR “community dwelling*” OR “community-dwelling*” OR “retirement communit*” OR “retirement cent*” OR “retirement life care cent*” OR “age in place” OR “aging in place” OR “ageing in place” OR “ages in place” OR “aged in place” OR “age at home” OR “aging at home” OR “ageing at home” OR “ages at home” OR “aged at home” OR “private residenc*” OR “domestic environment*” OR “home environment*” OR “reside at home” OR “resides at home” OR “residing at home” OR “resided at home” OR “assisted living” OR “living at home” OR “lives at home” OR “live at home” OR “lived at home” OR “community living” OR “congregate hous*”) OR (("Independent Living"[Mesh]) OR "Housing for the Elderly"[Mesh]) OR "Assisted Living Facilities"[Mesh]

AND

Terms for functional training

TIAB (“functional task*” OR “functional exercis*” OR “functional therap*” OR “functional skill*” OR “functional performance” OR “functional activit*” OR “functional occupation*” OR “functional train*” OR “functional retrain*” OR “functional rehab*” OR “functional intervention*” OR “functional strength task*” OR “functional strength exercise*” OR “functional strength therap*” OR “functional strength skill*” OR “functional strength perform*” OR “functional strength activit*” OR “functional strength occupation*” OR “functional strength train*” OR “functional strength retrain*” OR “functional strength rehab*” OR “functional strength intervention*” OR “functional oriented rehab” OR “functional-oriented rehab*” OR (functional AND specificity) OR “occupational task*” OR “occupational exercis*” OR “occupational skill*” OR “occupational performance” OR “occupational activit*” OR “occupational train*” OR “occupational retrain*” OR “occupational rehab*” OR “occupational intervention*” OR “occupational coordination” OR “occupational co-ordination” OR (occupational AND specificity) OR “chronic activity limitation*” OR “chronic limitation of activit*” OR “daily life task*” OR “daily life exercis*” OR “daily life therap*” OR “daily life skill*” OR “daily life performance” OR “daily life activit*” OR “daily life occupational” OR “daily life train*” OR “daily life retrain*” OR “daily life rehab*” OR “daily life intervention*” OR “daily life coordination” OR “daily life co-ordination” OR “daily lives task*” OR “daily lives exercis*” OR “daily lives therap*” OR “daily lives skill*” OR “daily lives performance” OR “daily lives activit*” OR “daily lives occupational” OR “daily lives train*” OR “daily lives retrain*” OR “daily lives rehab*” OR “daily lives intervention*” OR “daily lives coordination*” OR “daily lives co-ordination” OR “psychomotor task*” OR “psychomotor exercis*” OR “psychomotor therap*” OR “psychomotor skill*” OR “psychomotor performance” OR “psychomotor activit*” OR “psychomotor occupation*” OR “psychomotor train*” OR “psychomotor retrain*” OR “psychomotor rehab*” OR “psychomotor intervention*” OR (psychomotor AND specificity) OR “life integrat*” OR “lifestyle integrat*” OR “daily living task*” OR “daily living exercis*” OR “daily living therap*” OR “daily living skill*” OR “daily living performance” OR “daily living activit*” OR “daily living occupational” OR “daily living train*” OR “daily living retrain*” OR “daily living rehab*” OR “daily living intervention*” OR “daily living coordination” OR “daily living co-ordination” OR “daily task*” OR “daily activity task*” OR “daily activities task*” “daily activity performance” OR “daily activities performance” OR “daily activity occupation*” OR “daily activities occupation*” OR “daily activity train*” OR “daily activities train*” OR “daily activity retrain*” OR “daily activities retrain*” OR “daily activity rehab*” OR “daily activities rehab*” OR “daily activity intervention*” OR “daily activities intervention*” OR “daily activity coordination” OR “daily activity co-ordination” OR “daily activities coordination” OR “daily activities co-ordination” OR “ADAP” OR “everyday task*” OR “every day task*” OR “everyday activit*” OR “every day activit*” OR “every day occupational” OR “everyday occupational” OR “everyday living task*” OR “every day living task*” OR “everyday living exercis*” OR “every day living exercis*” OR “everyday living therap*” OR “every day living therap*” OR “everyday living skill*” OR “every day living skill*” OR “everyday living performance” OR “every day living performance” OR “every day living activit*” OR “everyday living activit*” OR “everyday living occupation*” OR “every day living occupation*” OR “everyday living train*” OR “every day living train*” OR “every day living retrain*” OR “everyday living train*” OR “every day living rehab*” OR “everyday living rehab*” OR “every day living intervention*” OR “everyday living intervention*” OR “every day living coordination” OR “everyday living coordination” OR “every day living co-ordination” OR “everyday living co-ordination” OR “every day life task*” OR “everyday life task*” OR “everyday life exercis*” OR “every day life exercis*” OR “every day life therap*” OR “everyday life therap*” OR “everyday life skill*” OR “every day life skill*” OR “every day life performance” OR “everyday life performance” OR “everyday life activit*” OR “every day life activit*” OR “every day life occupation*” OR “everyday life occupation*” OR “every day life train*” OR “everyday life train*” OR “every day life retrain*” OR “everyday life retrain*” OR “every day life rehab*” OR “everyday life rehab*” OR “everyday life intervention*” OR “every day life intervention*” OR “every day life coordination” OR “everyday life coordination” OR “every day life co-ordination” OR “everyday life co-ordination” OR “self care task*” OR “self-care task*” OR “self care exercis*” OR “self-care exercis*” OR “self care therap*” OR “self-care therap*” OR “self care skill*” OR “self-care skill*” OR “self care performance” OR “self-care performance” OR “self care activit*” OR “self-care activit*” OR “self care occupation*” OR “self-care occupation*” OR “self care train*” OR “self-care train*” OR “self care retrain*” OR “self-care retrain*” OR “self care rehab*” OR “self-care rehab*” OR “self care intervention*” OR “self-care intervention*”) OR (("Physical Functional Performance"[Mesh:NoExp])

OR

(shower* OR bathe OR bathes OR bathing OR “bathroom transfer*” OR “personal hygiene” OR “toilet hygiene” OR grooming OR dressing OR “getting dressed” OR laundry OR vacuuming OR hoovering OR ironing OR housekeep* OR “household management” OR “housework*” OR “managing house*” OR “manage house*” OR “managed house*” OR homemak* OR cleaning OR “purposeful movement*” OR “purposeful activit*” OR ADL OR ADLS OR IADL OR IADLS OR “activity of daily living” OR “activities of daily living”) OR ("Activities of Daily Living/therapeutic use"[Mesh]) AND (task* OR exercis* OR performance OR train* OR retrain* OR rehab* OR intervention* OR specificity)

NOT

TI: (protocol* OR conference* OR proceedings OR “book review” OR editorial)

AND

Limits: 2010 onwards; English

**SPORTDISCUS**

- Population: Older adults (65 years and over), age-related change only (no other conditions), community dwelling

Terms for older adults

Title/Abstract: (“older adult*” OR “old adult*” OR elder* OR “old people” OR “older people” OR “old person*” OR “older person*” OR “older communit*” OR "older population*” OR "senior people" OR "senior person*" OR “senior citizen*” OR "senior communit*” OR "senior population*” OR geriatric* OR frail* OR prefrail* OR “pre-frail*” OR “oldest old” OR “old age” OR “old aged” OR “older age” OR “older aged” OR “age related cognitive impairment*” OR “age-related cognitive impairment*” OR “ARCI” OR fragile OR fragility OR “aging adult*” OR “ageing adult*”) OR (DE "OLDER people's injuries" OR DE "OLDER people physiology" OR DE "HEALTH of older people" OR DE "PHYSICAL fitness for older people" OR DE "OLDER people -- Nutrition" OR DE "EXERCISE for older people" OR DE "MUSCLE aging" OR DE "INFLUENCE of age on ability" OR DE "AGING" OR DE "SPORTS for older people" OR DE "RETIREMENT" OR DE "RECREATION for older people" OR DE "PHYSICAL education for older people" OR DE "OLDER people" OR DE "OLDER athletes" OR DE "GERIATRICS" OR DE "RECREATIONAL therapy for older people" OR DE "EXERCISE therapy for older people")

AND

Terms for community dwelling

TIAB (“independent living” OR “living independently” OR “live independently” OR “lives independently” OR “community dwelling*” OR “community-dwelling*” OR “retirement communit*” OR “retirement cent*” OR “retirement life care cent*” OR “age in place” OR “aging in place” OR “ageing in place” OR “ages in place” OR “aged in place” OR “age at home” OR “aging at home” OR “ageing at home” OR “ages at home” OR “aged at home” OR “private residenc*” OR “domestic environment*” OR “home environment*” OR “reside at home” OR “resides at home” OR “residing at home” OR “resided at home” OR “assisted living” OR “living at home” OR “lives at home” OR “live at home” OR “lived at home” OR “community living” OR “congregate hous*”)

AND

Terms for functional training

TIAB (“functional task*” OR “functional exercis*” OR “functional therap*” OR “functional skill*” OR “functional performance” OR “functional activit*” OR “functional occupation*” OR “functional train*” OR “functional retrain*” OR “functional rehab*” OR “functional intervention*” OR “functional strength task*” OR “functional strength exercise*” OR “functional strength therap*” OR “functional strength skill*” OR “functional strength perform*” OR “functional strength activit*” OR “functional strength occupation*” OR “functional strength train*” OR “functional strength retrain*” OR “functional strength rehab*” OR “functional strength intervention*” OR “functional oriented rehab” OR “functional-oriented rehab*” OR (functional AND specificity) OR “occupational task*” OR “occupational exercis*” OR “occupational skill*” OR “occupational performance” OR “occupational activit*” OR “occupational train*” OR “occupational retrain*” OR “occupational rehab*” OR “occupational intervention*” OR “occupational coordination” OR “occupational co-ordination” OR (occupational AND specificity) OR “chronic activity limitation*” OR “chronic limitation of activit*” OR “daily life task*” OR “daily life exercis*” OR “daily life therap*” OR “daily life skill*” OR “daily life performance” OR “daily life activit*” OR “daily life occupational” OR “daily life train*” OR “daily life retrain*” OR “daily life rehab*” OR “daily life intervention*” OR “daily life coordination” OR “daily life co-ordination” OR “daily lives task*” OR “daily lives exercis*” OR “daily lives therap*” OR “daily lives skill*” OR “daily lives performance” OR “daily lives activit*” OR “daily lives occupational” OR “daily lives train*” OR “daily lives retrain*” OR “daily lives rehab*” OR “daily lives intervention*” OR “daily lives coordination*” OR “daily lives co-ordination” OR “psychomotor task*” OR “psychomotor exercis*” OR “psychomotor therap*” OR “psychomotor skill*” OR “psychomotor performance” OR “psychomotor activit*” OR “psychomotor occupation*” OR “psychomotor train*” OR “psychomotor retrain*” OR “psychomotor rehab*” OR “psychomotor intervention*” OR (psychomotor AND specificity) OR “life integrat*” OR “lifestyle integrat*” OR “daily living task*” OR “daily living exercis*” OR “daily living therap*” OR “daily living skill*” OR “daily living performance” OR “daily living activit*” OR “daily living occupational” OR “daily living train*” OR “daily living retrain*” OR “daily living rehab*” OR “daily living intervention*” OR “daily living coordination” OR “daily living co-ordination” OR “daily task*” OR “daily activity task*” OR “daily activities task*” “daily activity performance” OR “daily activities performance” OR “daily activity occupation*” OR “daily activities occupation*” OR “daily activity train*” OR “daily activities train*” OR “daily activity retrain*” OR “daily activities retrain*” OR “daily activity rehab*” OR “daily activities rehab*” OR “daily activity intervention*” OR “daily activities intervention*” OR “daily activity coordination” OR “daily activity co-ordination” OR “daily activities coordination” OR “daily activities co-ordination” OR “ADAP” OR “everyday task*” OR “every day task*” OR “everyday activit*” OR “every day activit*” OR “every day occupational” OR “everyday occupational” OR “everyday living task*” OR “every day living task*” OR “everyday living exercis*” OR “every day living exercis*” OR “everyday living therap*” OR “every day living therap*” OR “everyday living skill*” OR “every day living skill*” OR “everyday living performance” OR “every day living performance” OR “every day living activit*” OR “everyday living activit*” OR “everyday living occupation*” OR “every day living occupation*” OR “everyday living train*” OR “every day living train*” OR “every day living retrain*” OR “everyday living train*” OR “every day living rehab*” OR “everyday living rehab*” OR “every day living intervention*” OR “everyday living intervention*” OR “every day living coordination” OR “everyday living coordination” OR “every day living co-ordination” OR “everyday living co-ordination” OR “every day life task*” OR “everyday life task*” OR “everyday life exercis*” OR “every day life exercis*” OR “every day life therap*” OR “everyday life therap*” OR “everyday life skill*” OR “every day life skill*” OR “every day life performance” OR “everyday life performance” OR “everyday life activit*” OR “every day life activit*” OR “every day life occupation*” OR “everyday life occupation*” OR “every day life train*” OR “everyday life train*” OR “every day life retrain*” OR “everyday life retrain*” OR “every day life rehab*” OR “everyday life rehab*” OR “everyday life intervention*” OR “every day life intervention*” OR “every day life coordination” OR “everyday life coordination” OR “every day life co-ordination” OR “everyday life co-ordination” OR “self care task*” OR “self-care task*” OR “self care exercis*” OR “self-care exercis*” OR “self care therap*” OR “self-care therap*” OR “self care skill*” OR “self-care skill*” OR “self care performance” OR “self-care performance” OR “self care activit*” OR “self-care activit*” OR “self care occupation*” OR “self-care occupation*” OR “self care train*” OR “self-care train*” OR “self care retrain*” OR “self-care retrain*” OR “self care rehab*” OR “self-care rehab*” OR “self care intervention*” OR “self-care intervention*”) OR (DE "FUNCTIONAL independence measure" OR DE "ACTIVITIES of daily living training" OR DE "FUNCTIONAL training" OR DE "OCCUPATIONAL rehabilitation")

OR

(shower* OR bathe OR bathes OR bathing OR “bathroom transfer*” OR “personal hygiene” OR “toilet hygiene” OR grooming OR dressing OR “getting dressed” OR laundry OR vacuuming OR hoovering OR ironing OR housekeep* OR “household management” OR “housework*” OR “managing house*” OR “manage house*” OR “managed house*” OR homemak* OR cleaning OR “purposeful movement*” OR “purposeful activit*” OR ADL OR ADLS OR IADL OR IADLS OR “activity of daily living” OR “activities of daily living”) OR (DE "ACTIVITIES of daily living") AND (task* OR exercis* OR performance OR train* OR retrain*OR rehab* OR intervention* OR specificity)

NOT

TI: (protocol* OR conference* OR proceedings OR “book review” OR editorial)

AND

Limits: 2010-2021; English; Academic Journals

**PsycINFO**

- Population: Older adults (65 years and over), age-related change only (no other conditions), community dwelling

Terms for older adults

Title/Abstract: (“older adult*” OR “old adult*” OR elder* OR “old people” OR “older people” OR “old person*” OR “older person*” OR “older communit*” OR "older population*” OR "senior people" OR "senior person*" OR “senior citizen*” OR "senior communit*” OR "senior population*” OR geriatric* OR frail* OR prefrail* OR “pre-frail*” OR “oldest old” OR “old age” OR “old aged” OR “older age” OR “older aged” OR “age related cognitive impairment*” OR “age-related cognitive impairment*” OR “ARCI” OR fragile OR fragility OR “aging adult*” OR “ageing adult*”) OR (DE "OLDER people's injuries" OR DE "OLDER people physiology" OR DE "HEALTH of older people" OR DE "PHYSICAL fitness for older people" OR DE "OLDER people -- Nutrition" OR DE "EXERCISE for older people" OR DE "MUSCLE aging" OR DE "INFLUENCE of age on ability" OR DE "AGING" OR DE "SPORTS for older people" OR DE "RETIREMENT" OR DE "RECREATION for older people" OR DE "PHYSICAL education for older people" OR DE "OLDER people" OR DE "OLDER athletes" OR DE "GERIATRICS" OR DE "RECREATIONAL therapy for older people" OR DE "EXERCISE therapy for older people")

AND

Terms for community dwelling

TIAB (“independent living” OR “living independently” OR “live independently” OR “lives independently” OR “community dwelling*” OR “community-dwelling*” OR “retirement communit*” OR “retirement cent*” OR “retirement life care cent*” OR “age in place” OR “aging in place” OR “ageing in place” OR “ages in place” OR “aged in place” OR “age at home” OR “aging at home” OR “ageing at home” OR “ages at home” OR “aged at home” OR “private residenc*” OR “domestic environment*” OR “home environment*” OR “reside at home” OR “resides at home” OR “residing at home” OR “resided at home” OR “assisted living” OR “living at home” OR “lives at home” OR “live at home” OR “lived at home” OR “community living” OR “congregate hous*”)

AND

Terms for functional training

TIAB (“functional task*” OR “functional exercis*” OR “functional therap*” OR “functional skill*” OR “functional performance” OR “functional activit*” OR “functional occupation*” OR “functional train*” OR “functional retrain*” OR “functional rehab*” OR “functional intervention*” OR “functional strength task*” OR “functional strength exercise*” OR “functional strength therap*” OR “functional strength skill*” OR “functional strength perform*” OR “functional strength activit*” OR “functional strength occupation*” OR “functional strength train*” OR “functional strength retrain*” OR “functional strength rehab*” OR “functional strength intervention*” OR “functional oriented rehab” OR “functional-oriented rehab*” OR (functional AND specificity) OR “occupational task*” OR “occupational exercis*” OR “occupational skill*” OR “occupational performance” OR “occupational activit*” OR “occupational train*” OR “occupational retrain*” OR “occupational rehab*” OR “occupational intervention*” OR “occupational coordination” OR “occupational co-ordination” OR (occupational AND specificity) OR “chronic activity limitation*” OR “chronic limitation of activit*” OR “daily life task*” OR “daily life exercis*” OR “daily life therap*” OR “daily life skill*” OR “daily life performance” OR “daily life activit*” OR “daily life occupational” OR “daily life train*” OR “daily life retrain*” OR “daily life rehab*” OR “daily life intervention*” OR “daily life coordination” OR “daily life co-ordination” OR “daily lives task*” OR “daily lives exercis*” OR “daily lives therap*” OR “daily lives skill*” OR “daily lives performance” OR “daily lives activit*” OR “daily lives occupational” OR “daily lives train*” OR “daily lives retrain*” OR “daily lives rehab*” OR “daily lives intervention*” OR “daily lives coordination*” OR “daily lives co-ordination” OR “psychomotor task*” OR “psychomotor exercis*” OR “psychomotor therap*” OR “psychomotor skill*” OR “psychomotor performance” OR “psychomotor activit*” OR “psychomotor occupation*” OR “psychomotor train*” OR “psychomotor retrain*” OR “psychomotor rehab*” OR “psychomotor intervention*” OR (psychomotor AND specificity) OR “life integrat*” OR “lifestyle integrat*” OR “daily living task*” OR “daily living exercis*” OR “daily living therap*” OR “daily living skill*” OR “daily living performance” OR “daily living activit*” OR “daily living occupational” OR “daily living train*” OR “daily living retrain*” OR “daily living rehab*” OR “daily living intervention*” OR “daily living coordination” OR “daily living co-ordination” OR “daily task*” OR “daily activity task*” OR “daily activities task*” “daily activity performance” OR “daily activities performance” OR “daily activity occupation*” OR “daily activities occupation*” OR “daily activity train*” OR “daily activities train*” OR “daily activity retrain*” OR “daily activities retrain*” OR “daily activity rehab*” OR “daily activities rehab*” OR “daily activity intervention*” OR “daily activities intervention*” OR “daily activity coordination” OR “daily activity co-ordination” OR “daily activities coordination” OR “daily activities co-ordination” OR “ADAP” OR “everyday task*” OR “every day task*” OR “everyday activit*” OR “every day activit*” OR “every day occupational” OR “everyday occupational” OR “everyday living task*” OR “every day living task*” OR “everyday living exercis*” OR “every day living exercis*” OR “everyday living therap*” OR “every day living therap*” OR “everyday living skill*” OR “every day living skill*” OR “everyday living performance” OR “every day living performance” OR “every day living activit*” OR “everyday living activit*” OR “everyday living occupation*” OR “every day living occupation*” OR “everyday living train*” OR “every day living train*” OR “every day living retrain*” OR “everyday living train*” OR “every day living rehab*” OR “everyday living rehab*” OR “every day living intervention*” OR “everyday living intervention*” OR “every day living coordination” OR “everyday living coordination” OR “every day living co-ordination” OR “everyday living co-ordination” OR “every day life task*” OR “everyday life task*” OR “everyday life exercis*” OR “every day life exercis*” OR “every day life therap*” OR “everyday life therap*” OR “everyday life skill*” OR “every day life skill*” OR “every day life performance” OR “everyday life performance” OR “everyday life activit*” OR “every day life activit*” OR “every day life occupation*” OR “everyday life occupation*” OR “every day life train*” OR “everyday life train*” OR “every day life retrain*” OR “everyday life retrain*” OR “every day life rehab*” OR “everyday life rehab*” OR “everyday life intervention*” OR “every day life intervention*” OR “every day life coordination” OR “everyday life coordination” OR “every day life co-ordination” OR “everyday life co-ordination” OR “self care task*” OR “self-care task*” OR “self care exercis*” OR “self-care exercis*” OR “self care therap*” OR “self-care therap*” OR “self care skill*” OR “self-care skill*” OR “self care performance” OR “self-care performance” OR “self care activit*” OR “self-care activit*” OR “self care occupation*” OR “self-care occupation*” OR “self care train*” OR “self-care train*” OR “self care retrain*” OR “self-care retrain*” OR “self care rehab*” OR “self-care rehab*” OR “self care intervention*” OR “self-care intervention*”) OR (DE "FUNCTIONAL independence measure" OR DE "ACTIVITIES of daily living training" OR DE "FUNCTIONAL training" OR DE "OCCUPATIONAL rehabilitation")

OR

(shower* OR bathe OR bathes OR bathing OR “bathroom transfer*” OR “personal hygiene” OR “toilet hygiene” OR grooming OR dressing OR “getting dressed” OR laundry OR vacuuming OR hoovering OR ironing OR housekeep* OR “household management” OR “housework*” OR “managing house*” OR “manage house*” OR “managed house*” OR homemak* OR cleaning OR “purposeful movement*” OR “purposeful activit*” OR ADL OR ADLS OR IADL OR IADLS OR “activity of daily living” OR “activities of daily living”) OR (DE "Activities of Daily Living") AND (task* OR exercis* OR performance OR train* OR retrain*OR rehab* OR intervention* OR specificity)

NOT

TI: (protocol* OR conference* OR proceedings OR “book review” OR editorial)

AND

**PEDRO**

Abstract and Title: “functional training” AND elderly OR “older adult” OR “older adults” OR “older adulthood” OR geriatric OR geriatrics OR aging OR ageing OR “independent living” OR “community living”

Since 2010

**REHABDATA**

Title/Abstract: “functional training” AND elderly OR “older adult” OR “older adults” OR “older adulthood” OR geriatric OR geriatrics

Limits 2010 – 2021; English

**PsycINFO**

Terms for older adults

Title/Abstract: (“older adult*” OR “old adult*” OR elder* OR “old people” OR “older people” OR “old person*” OR “older person*” OR “older communit*” OR "older population*” OR "senior people" OR "senior person*" OR “senior citizen*” OR "senior communit*” OR "senior population*” OR geriatric* OR frail* OR prefrail* OR “pre-frail*” OR “oldest old” OR “old age” OR “old aged” OR “older age” OR “older aged” OR “age related cognitive impairment*” OR “age-related cognitive impairment*” OR “ARCI” OR fragile OR fragility OR “aging adult*” OR “ageing adult*”) OR (DE "Geriatric Patients" OR DE "Older Adulthood" OR DE "Aging" OR DE "Cognitive Aging" OR DE "Healthy Aging" OR DE "Gerontology")

AND

Terms for community dwelling

TIAB (“independent living” OR “living independently” OR “live independently” OR “lives independently” OR “community dwelling*” OR “community-dwelling*” OR “retirement communit*” OR “retirement cent*” OR “retirement life care cent*” OR “age in place” OR “aging in place” OR “ageing in place” OR “ages in place” OR “aged in place” OR “age at home” OR “aging at home” OR “ageing at home” OR “ages at home” OR “aged at home” OR “private residenc*” OR “domestic environment*” OR “home environment*” OR “reside at home” OR “resides at home” OR “residing at home” OR “resided at home” OR “assisted living” OR “living at home” OR “lives at home” OR “live at home” OR “lived at home” OR “community living” OR “congregate hous*”) OR (DE "Aging in Place" OR DE "Independent Living Programs" OR DE "Living Alone" OR DE "Retirement Communities" OR DE "Assisted Living")

AND

Terms for functional training

TIAB (“functional task*” OR “functional exercis*” OR “functional therap*” OR “functional skill*” OR “functional performance” OR “functional activit*” OR “functional occupation*” OR “functional train*” OR “functional retrain*” OR “functional rehab*” OR “functional intervention*” OR “functional strength task*” OR “functional strength exercise*” OR “functional strength therap*” OR “functional strength skill*” OR “functional strength perform*” OR “functional strength activit*” OR “functional strength occupation*” OR “functional strength train*” OR “functional strength retrain*” OR “functional strength rehab*” OR “functional strength intervention*” OR “functional oriented rehab” OR “functional-oriented rehab*” OR (functional AND specificity) OR “occupational task*” OR “occupational exercis*” OR “occupational skill*” OR “occupational performance” OR “occupational activit*” OR “occupational train*” OR “occupational retrain*” OR “occupational rehab*” OR “occupational intervention*” OR “occupational coordination” OR “occupational co-ordination” OR (occupational AND specificity) OR “chronic activity limitation*” OR “chronic limitation of activit*” OR “daily life task*” OR “daily life exercis*” OR “daily life therap*” OR “daily life skill*” OR “daily life performance” OR “daily life activit*” OR “daily life occupational” OR “daily life train*” OR “daily life retrain*” OR “daily life rehab*” OR “daily life intervention*” OR “daily life coordination” OR “daily life co-ordination” OR “daily lives task*” OR “daily lives exercis*” OR “daily lives therap*” OR “daily lives skill*” OR “daily lives performance” OR “daily lives activit*” OR “daily lives occupational” OR “daily lives train*” OR “daily lives retrain*” OR “daily lives rehab*” OR “daily lives intervention*” OR “daily lives coordination*” OR “daily lives co-ordination” OR “psychomotor task*” OR “psychomotor exercis*” OR “psychomotor therap*” OR “psychomotor skill*” OR “psychomotor performance” OR “psychomotor activit*” OR “psychomotor occupation*” OR “psychomotor train*” OR “psychomotor retrain*” OR “psychomotor rehab*” OR “psychomotor intervention*” OR (psychomotor AND specificity) OR “life integrat*” OR “lifestyle integrat*” OR “daily living task*” OR “daily living exercis*” OR “daily living therap*” OR “daily living skill*” OR “daily living performance” OR “daily living activit*” OR “daily living occupational” OR “daily living train*” OR “daily living retrain*” OR “daily living rehab*” OR “daily living intervention*” OR “daily living coordination” OR “daily living co-ordination” OR “daily task*” OR “daily activity task*” OR “daily activities task*” “daily activity performance” OR “daily activities performance” OR “daily activity occupation*” OR “daily activities occupation*” OR “daily activity train*” OR “daily activities train*” OR “daily activity retrain*” OR “daily activities retrain*” OR “daily activity rehab*” OR “daily activities rehab*” OR “daily activity intervention*” OR “daily activities intervention*” OR “daily activity coordination” OR “daily activity co-ordination” OR “daily activities coordination” OR “daily activities co-ordination” OR “ADAP” OR “everyday task*” OR “every day task*” OR “everyday activit*” OR “every day activit*” OR “every day occupational” OR “everyday occupational” OR “everyday living task*” OR “every day living task*” OR “everyday living exercis*” OR “every day living exercis*” OR “everyday living therap*” OR “every day living therap*” OR “everyday living skill*” OR “every day living skill*” OR “everyday living performance” OR “every day living performance” OR “every day living activit*” OR “everyday living activit*” OR “everyday living occupation*” OR “every day living occupation*” OR “everyday living train*” OR “every day living train*” OR “every day living retrain*” OR “everyday living train*” OR “every day living rehab*” OR “everyday living rehab*” OR “every day living intervention*” OR “everyday living intervention*” OR “every day living coordination” OR “everyday living coordination” OR “every day living co-ordination” OR “everyday living co-ordination” OR “every day life task*” OR “everyday life task*” OR “everyday life exercis*” OR “every day life exercis*” OR “every day life therap*” OR “everyday life therap*” OR “everyday life skill*” OR “every day life skill*” OR “every day life performance” OR “everyday life performance” OR “everyday life activit*” OR “every day life activit*” OR “every day life occupation*” OR “everyday life occupation*” OR “every day life train*” OR “everyday life train*” OR “every day life retrain*” OR “everyday life retrain*” OR “every day life rehab*” OR “everyday life rehab*” OR “everyday life intervention*” OR “every day life intervention*” OR “every day life coordination” OR “everyday life coordination” OR “every day life co-ordination” OR “everyday life co-ordination” OR “self care task*” OR “self-care task*” OR “self care exercis*” OR “self-care exercis*” OR “self care therap*” OR “self-care therap*” OR “self care skill*” OR “self-care skill*” OR “self care performance” OR “self-care performance” OR “self care activit*” OR “self-care activit*” OR “self care occupation*” OR “self-care occupation*” OR “self care train*” OR “self-care train*” OR “self care retrain*” OR “self-care retrain*” OR “self care rehab*” OR “self-care rehab*” OR “self care intervention*” OR “self-care intervention*”)

OR

(shower* OR bathe OR bathes OR bathing OR “bathroom transfer*” OR “personal hygiene” OR “toilet hygiene” OR grooming OR dressing OR “getting dressed” OR laundry OR vacuuming OR hoovering OR ironing OR housekeep* OR “household management” OR “housework*” OR “managing house*” OR “manage house*” OR “managed house*” OR homemak* OR cleaning OR “purposeful movement*” OR “purposeful activit*” OR ADL OR ADLS OR IADL OR IADLS OR “activity of daily living” OR “activities of daily living”) OR (DE "Activities of Daily Living") AND (task* OR exercis* OR performance OR train* OR retrain*OR rehab* OR intervention* OR specificity)

NOT

TI: (protocol* OR conference* OR proceedings OR “book review” OR editorial)

AND

Limits: 2010 onward; English; Academic Journals OR Dissertations

**Psychology and Behavioral Sciences Collection**

- Population: Older adults (65 years and over), age-related change only (no other conditions), community dwelling

Terms for older adults

Title/Abstract: (“older adult*” OR “old adult*” OR elder* OR “old people” OR “older people” OR “old person*” OR “older person*” OR “older communit*” OR "older population*” OR "senior people" OR "senior person*" OR “senior citizen*” OR "senior communit*” OR "senior population*” OR geriatric* OR frail* OR prefrail* OR “pre-frail*” OR “oldest old” OR “old age” OR “old aged” OR “older age” OR “older aged” OR “age related cognitive impairment*” OR “age-related cognitive impairment*” OR “ARCI” OR fragile OR fragility OR “aging adult*” OR “ageing adult*”) OR (DE "CENTENARIANS" OR DE "EXERCISE for older people" OR DE "MENTALLY ill older people" OR DE "MINORITY older people" OR DE "OLDER men" OR DE "OLDER women" OR DE "PHYSICAL fitness for older people" OR DE "ACTIVE aging" OR DE "AGING" OR DE "RETIREES" OR DE "OLDER people's injuries" OR DE "HEALTH of older people" OR DE "ELDERLY poor" OR DE "RURAL elderly" OR DE "URBAN elderly" OR DE "OLDER people" OR DE "OLDER patients" OR DE "AT-risk older people" OR DE "OLD-old" OR DE "FRAIL elderly" OR DE "FRAIL elderly diseases" OR DE "OLD age" OR DE "COGNITION in old age" OR DE "PREVENTION of falls in old age" OR DE "MEMORY disorders in old age" OR DE "COGNITION disorders in old age" OR DE "SELF-reliance in old age" OR DE "SELF-efficacy in old age" OR DE "PAIN in old age" OR DE "NUTRITION disorders in old age" OR DE "MUSCULOSKELETAL diseases in old age" OR DE "MOVEMENT disorders in old age" OR DE "GAIT disorders in old age" OR DE "DISEASES in older people" OR DE "BONE fractures in old age" OR DE "ACCIDENTAL falls in old age" OR "FUNCTIONAL loss in older people")

AND

Terms for community dwelling

TIAB (“independent living” OR “living independently” OR “live independently” OR “lives independently” OR “community dwelling*” OR “community-dwelling*” OR “retirement communit*” OR “retirement cent*” OR “retirement life care cent*” OR “age in place” OR “aging in place” OR “ageing in place” OR “ages in place” OR “aged in place” OR “age at home” OR “aging at home” OR “ageing at home” OR “ages at home” OR “aged at home” OR “private residenc*” OR “domestic environment*” OR “home environment*” OR “reside at home” OR “resides at home” OR “residing at home” OR “resided at home” OR “assisted living” OR “living at home” OR “lives at home” OR “live at home” OR “lived at home” OR “community living” OR “congregate hous*”) OR (DE "RETIREMENT communities" OR DE "INDEPENDENT living" OR DE "CONGREGATE housing" OR DE "AGING in place" OR DE "SENIOR housing"))

AND

Terms for functional training

TIAB (“functional task*” OR “functional exercis*” OR “functional therap*” OR “functional skill*” OR “functional performance” OR “functional activit*” OR “functional occupation*” OR “functional train*” OR “functional retrain*” OR “functional rehab*” OR “functional intervention*” OR “functional strength task*” OR “functional strength exercise*” OR “functional strength therap*” OR “functional strength skill*” OR “functional strength perform*” OR “functional strength activit*” OR “functional strength occupation*” OR “functional strength train*” OR “functional strength retrain*” OR “functional strength rehab*” OR “functional strength intervention*” OR “functional oriented rehab” OR “functional-oriented rehab*” OR (functional AND specificity) OR “occupational task*” OR “occupational exercis*” OR “occupational skill*” OR “occupational performance” OR “occupational activit*” OR “occupational train*” OR “occupational retrain*” OR “occupational rehab*” OR “occupational intervention*” OR “occupational coordination” OR “occupational co-ordination” OR (occupational AND specificity) OR “chronic activity limitation*” OR “chronic limitation of activit*” OR “daily life task*” OR “daily life exercis*” OR “daily life therap*” OR “daily life skill*” OR “daily life performance” OR “daily life activit*” OR “daily life occupational” OR “daily life train*” OR “daily life retrain*” OR “daily life rehab*” OR “daily life intervention*” OR “daily life coordination” OR “daily life co-ordination” OR “daily lives task*” OR “daily lives exercis*” OR “daily lives therap*” OR “daily lives skill*” OR “daily lives performance” OR “daily lives activit*” OR “daily lives occupational” OR “daily lives train*” OR “daily lives retrain*” OR “daily lives rehab*” OR “daily lives intervention*” OR “daily lives coordination*” OR “daily lives co-ordination” OR “psychomotor task*” OR “psychomotor exercis*” OR “psychomotor therap*” OR “psychomotor skill*” OR “psychomotor performance” OR “psychomotor activit*” OR “psychomotor occupation*” OR “psychomotor train*” OR “psychomotor retrain*” OR “psychomotor rehab*” OR “psychomotor intervention*” OR (psychomotor AND specificity) OR “life integrat*” OR “lifestyle integrat*” OR “daily living task*” OR “daily living exercis*” OR “daily living therap*” OR “daily living skill*” OR “daily living performance” OR “daily living activit*” OR “daily living occupational” OR “daily living train*” OR “daily living retrain*” OR “daily living rehab*” OR “daily living intervention*” OR “daily living coordination” OR “daily living co-ordination” OR “daily task*” OR “daily activity task*” OR “daily activities task*” “daily activity performance” OR “daily activities performance” OR “daily activity occupation*” OR “daily activities occupation*” OR “daily activity train*” OR “daily activities train*” OR “daily activity retrain*” OR “daily activities retrain*” OR “daily activity rehab*” OR “daily activities rehab*” OR “daily activity intervention*” OR “daily activities intervention*” OR “daily activity coordination” OR “daily activity co-ordination” OR “daily activities coordination” OR “daily activities co-ordination” OR “ADAP” OR “everyday task*” OR “every day task*” OR “everyday activit*” OR “every day activit*” OR “every day occupational” OR “everyday occupational” OR “everyday living task*” OR “every day living task*” OR “everyday living exercis*” OR “every day living exercis*” OR “everyday living therap*” OR “every day living therap*” OR “everyday living skill*” OR “every day living skill*” OR “everyday living performance” OR “every day living performance” OR “every day living activit*” OR “everyday living activit*” OR “everyday living occupation*” OR “every day living occupation*” OR “everyday living train*” OR “every day living train*” OR “every day living retrain*” OR “everyday living train*” OR “every day living rehab*” OR “everyday living rehab*” OR “every day living intervention*” OR “everyday living intervention*” OR “every day living coordination” OR “everyday living coordination” OR “every day living co-ordination” OR “everyday living co-ordination” OR “every day life task*” OR “everyday life task*” OR “everyday life exercis*” OR “every day life exercis*” OR “every day life therap*” OR “everyday life therap*” OR “everyday life skill*” OR “every day life skill*” OR “every day life performance” OR “everyday life performance” OR “everyday life activit*” OR “every day life activit*” OR “every day life occupation*” OR “everyday life occupation*” OR “every day life train*” OR “everyday life train*” OR “every day life retrain*” OR “everyday life retrain*” OR “every day life rehab*” OR “everyday life rehab*” OR “everyday life intervention*” OR “every day life intervention*” OR “every day life coordination” OR “everyday life coordination” OR “every day life co-ordination” OR “everyday life co-ordination” OR “self care task*” OR “self-care task*” OR “self care exercis*” OR “self-care exercis*” OR “self care therap*” OR “self-care therap*” OR “self care skill*” OR “self-care skill*” OR “self care performance” OR “self-care performance” OR “self care activit*” OR “self-care activit*” OR “self care occupation*” OR “self-care occupation*” OR “self care train*” OR “self-care train*” OR “self care retrain*” OR “self-care retrain*” OR “self care rehab*” OR “self-care rehab*” OR “self care intervention*” OR “self-care intervention*”) OR DE "ACTIVITIES of daily living training" OR DE "FUNCTIONAL training"

OR

(shower* OR bathe OR bathes OR bathing OR “bathroom transfer*” OR “personal hygiene” OR “toilet hygiene” OR grooming OR dressing OR “getting dressed” OR laundry OR vacuuming OR hoovering OR ironing OR housekeep* OR “household management” OR “housework*” OR “managing house*” OR “manage house*” OR “managed house*” OR homemak* OR cleaning OR “purposeful movement*” OR “purposeful activit*” OR ADL OR ADLS OR IADL OR IADLS OR “activity of daily living” OR “activities of daily living”) OR (DE "ACTIVITIES of daily living")) AND (task* OR exercis* OR performance OR train* OR retrain*OR rehab* OR intervention* OR specificity)

NOT

TI: (protocol* OR conference* OR proceedings OR “book review” OR editorial)

AND

Limits: 2010-onward; English; All Academic Journals

**EMBASE**

(task*:ti,ab OR exercis*:ti,ab OR performance:ti,ab OR train*:ti,ab OR retrain*:ti,ab OR rehab*:ti,ab OR intervention*:ti,ab OR specificity:ti,ab) AND

(shower*:ti,ab OR bathe:ti,ab OR bathes:ti,ab OR bathing:ti,ab OR ‘bathroom transfer*’:ti,ab OR ‘personal hygiene’:ti,ab OR ‘toilet hygiene’:ti,ab OR grooming:ti,ab OR dressing:ti,ab OR ‘getting dressed’:ti,ab OR laundry:ti,ab OR vacuuming:ti,ab OR hoovering:ti,ab OR ironing:ti,ab OR housekeep*:ti,ab OR ‘household management’:ti,ab OR ‘housework*’:ti,ab OR ‘managing house*’:ti,ab OR ‘manage house*’:ti,ab OR ‘managed house*’:ti,ab OR homemak*:ti,ab OR cleaning:ti,ab OR ‘purposeful movement*’:ti,ab OR ‘purposeful activit*’:ti,ab OR ADL:ti,ab OR ADLS:ti,ab OR IADL:ti,ab OR IADLS:ti,ab OR ‘activity of daily living’:ti,ab OR ‘activities of daily living’:ti,ab) OR 'daily life activity'/de)

OR (‘functional task*’:ti,ab OR ‘functional exercis*’:ti,ab OR ‘functional therap*’:ti,ab OR ‘functional skill*’:ti,ab OR ‘functional performance’:ti,ab OR ‘functional activit*’:ti,ab OR ‘functional occupation*’:ti,ab OR ‘functional train*’:ti,ab OR ‘functional retrain*’:ti,ab OR ‘functional rehab*’:ti,ab OR ‘functional intervention*’:ti,ab OR ‘functional strength task*’:ti,ab OR ‘functional strength exercise*’:ti,ab OR ‘functional strength therap*’:ti,ab OR ‘functional strength skill*’:ti,ab OR ‘functional strength perform*’:ti,ab OR ‘functional strength activit*’:ti,ab OR ‘functional strength occupation*’:ti,ab OR ‘functional strength train*’:ti,ab OR ‘functional strength retrain*’:ti,ab OR ‘functional strength rehab*’:ti,ab OR ‘functional strength intervention*’:ti,ab OR ‘functional oriented rehab’:ti,ab OR ‘functional-oriented rehab*’:ti,ab OR (functional:ti,ab AND specificity:ti,ab) OR ‘occupational task*’:ti,ab OR ‘occupational exercis*’:ti,ab OR ‘occupational skill*’:ti,ab OR ‘occupational performance’:ti,ab OR ‘occupational activit*’:ti,ab OR ‘occupational train*’:ti,ab OR ‘occupational retrain*’:ti,ab OR ‘occupational rehab*’:ti,ab OR ‘occupational intervention*’:ti,ab OR ‘occupational coordination’:ti,ab OR ‘occupational co-ordination’:ti,ab OR (occupational:ti,ab AND specificity:ti,ab) OR ‘chronic activity limitation*’:ti,ab OR ‘chronic limitation of activit*’:ti,ab OR ‘daily life task*’:ti,ab OR ‘daily life exercis*’:ti,ab OR ‘daily life therap*’:ti,ab OR ‘daily life skill*’:ti,ab OR ‘daily life performance’:ti,ab OR ‘daily life activit*’:ti,ab OR ‘daily life occupational’:ti,ab OR ‘daily life train*’:ti,ab OR ‘daily life retrain*’:ti,ab OR ‘daily life rehab*’:ti,ab OR ‘daily life intervention*’:ti,ab OR ‘daily life coordination’:ti,ab OR ‘daily life co-ordination’:ti,ab OR ‘daily lives task*’:ti,ab OR ‘daily lives exercis*’:ti,ab OR ‘daily lives therap*’:ti,ab OR ‘daily lives skill*’:ti,ab OR ‘daily lives performance’:ti,ab OR ‘daily lives activit*’:ti,ab OR ‘daily lives occupational’:ti,ab OR ‘daily lives train*’:ti,ab OR ‘daily lives retrain*’:ti,ab OR ‘daily lives rehab*’:ti,ab OR ‘daily lives intervention*’:ti,ab OR ‘daily lives coordination*’:ti,ab OR ‘daily lives co-ordination’:ti,ab OR ‘psychomotor task*’:ti,ab OR ‘psychomotor exercis*’:ti,ab OR ‘psychomotor therap*’:ti,ab OR ‘psychomotor skill*’:ti,ab OR ‘psychomotor performance’:ti,ab OR ‘psychomotor activit*’:ti,ab OR ‘psychomotor occupation*’:ti,ab OR ‘psychomotor train*’:ti,ab OR ‘psychomotor retrain*’:ti,ab OR ‘psychomotor rehab*’:ti,ab OR ‘psychomotor intervention*’:ti,ab OR (psychomotor:ti,ab AND specificity:ti,ab) OR ‘life integrat*’:ti,ab OR ‘lifestyle integrat*’:ti,ab OR ‘daily living task*’:ti,ab OR ‘daily living exercis*’:ti,ab OR ‘daily living therap*’:ti,ab OR ‘daily living skill*’:ti,ab OR ‘daily living performance’:ti,ab OR ‘daily living activit*’:ti,ab OR ‘daily living occupational’:ti,ab OR ‘daily living train*’:ti,ab OR ‘daily living retrain*’:ti,ab OR ‘daily living rehab*’:ti,ab OR ‘daily living intervention*’:ti,ab OR ‘daily living coordination’:ti,ab OR ‘daily living co-ordination’:ti,ab OR ‘daily task*’:ti,ab OR ‘daily activity task*’:ti,ab OR ‘daily activities task*’:ti,ab OR ‘daily activity performance’:ti,ab OR ‘daily activities performance’:ti,ab OR ‘daily activity occupation*’:ti,ab OR ‘daily activities occupation*’:ti,ab OR ‘daily activity train*’:ti,ab OR ‘daily activities train*’:ti,ab OR ‘daily activity retrain*’:ti,ab OR ‘daily activities retrain*’:ti,ab OR ‘daily activity rehab*’:ti,ab OR ‘daily activities rehab*’:ti,ab OR ‘daily activity intervention*’:ti,ab OR ‘daily activities intervention*’:ti,ab OR ‘daily activity coordination’:ti,ab OR ‘daily activity co-ordination’:ti,ab OR ‘daily activities coordination’:ti,ab OR ‘daily activities co-ordination’:ti,ab OR ‘ADAP’:ti,ab OR ‘everyday task*’:ti,ab OR ‘every day task*’:ti,ab OR ‘everyday activit*’:ti,ab OR ‘every day activit*’:ti,ab OR ‘every day occupational’:ti,ab OR ‘everyday occupational’:ti,ab OR ‘everyday living task*’:ti,ab OR ‘every day living task*’:ti,ab OR ‘everyday living exercis*’:ti,ab OR ‘every day living exercis*’:ti,ab OR ‘everyday living therap*’:ti,ab OR ‘every day living therap*’:ti,ab OR ‘everyday living skill*’:ti,ab OR ‘every day living skill*’:ti,ab OR ‘everyday living performance’:ti,ab OR ‘every day living performance’:ti,ab OR ‘every day living activit*’:ti,ab OR ‘everyday living activit*’:ti,ab OR ‘everyday living occupation*’:ti,ab OR ‘every day living occupation*’:ti,ab OR ‘everyday living train*’:ti,ab OR ‘every day living train*’:ti,ab OR ‘every day living retrain*’:ti,ab OR ‘everyday living train*’:ti,ab OR ‘every day living rehab*’:ti,ab OR ‘everyday living rehab*’:ti,ab OR ‘every day living intervention*’:ti,ab OR ‘everyday living intervention*’:ti,ab OR ‘every day living coordination’:ti,ab OR ‘everyday living coordination’:ti,ab OR ‘every day living co-ordination’:ti,ab OR ‘everyday living co-ordination’:ti,ab OR ‘every day life task*’:ti,ab OR ‘everyday life task*’:ti,ab OR ‘everyday life exercis*’:ti,ab OR ‘every day life exercis*’:ti,ab OR ‘every day life therap*’:ti,ab OR ‘everyday life therap*’:ti,ab OR ‘everyday life skill*’:ti,ab OR ‘every day life skill*’:ti,ab OR ‘every day life performance’:ti,ab OR ‘everyday life performance’:ti,ab OR ‘everyday life activit*’:ti,ab OR ‘every day life activit*’:ti,ab OR ‘every day life occupation*’:ti,ab OR ‘everyday life occupation*’:ti,ab OR ‘every day life train*’:ti,ab OR ‘everyday life train*’:ti,ab OR ‘every day life retrain*’:ti,ab OR ‘everyday life retrain*’:ti,ab OR ‘every day life rehab*’:ti,ab OR ‘everyday life rehab*’:ti,ab OR ‘everyday life intervention*’:ti,ab OR ‘every day life intervention*’:ti,ab OR ‘every day life coordination’:ti,ab OR ‘everyday life coordination’:ti,ab OR ‘every day life co-ordination’:ti,ab OR ‘everyday life co-ordination’:ti,ab OR ‘self care task*’:ti,ab OR ‘self-care task*’:ti,ab OR ‘self care exercis*’:ti,ab OR ‘self-care exercis*’:ti,ab OR ‘self care therap*’:ti,ab OR ‘self-care therap*’:ti,ab OR ‘self care skill*’:ti,ab OR ‘self-care skill*’:ti,ab OR ‘self care performance’:ti,ab OR ‘self-care performance’:ti,ab OR ‘self care activit*’:ti,ab OR ‘self-care activit*’:ti,ab OR ‘self care occupation*’:ti,ab OR ‘self-care occupation*’:ti,ab OR ‘self care train*’:ti,ab OR ‘self-care train*’:ti,ab OR ‘self care retrain*’:ti,ab OR ‘self-care retrain*’:ti,ab OR ‘self care rehab*’:ti,ab OR ‘self-care rehab*’:ti,ab OR ‘self care intervention*’:ti,ab OR ‘self-care intervention*’:ti,ab) OR ('functional training'/de)

AND

(((‘older adult*’:ti,ab OR ‘old adult*’:ti,ab OR elder*:ti,ab OR ‘old people’:ti,ab OR ‘older people’:ti,ab OR ‘old person*’:ti,ab OR ‘older person*’:ti,ab OR ‘older communit*’:ti,ab OR ‘older population*’:ti,ab OR ‘senior people’:ti,ab OR ‘senior person*’:ti,ab OR ‘senior citizen*’:ti,ab OR ‘senior communit*’:ti,ab OR ‘senior population*’:ti,ab OR geriatric*:ti,ab OR frail*:ti,ab OR prefrail*:ti,ab OR ‘pre-frail*’:ti,ab OR ‘oldest old’:ti,ab OR ‘old age’:ti,ab OR ‘old aged’:ti,ab OR ‘older age’:ti,ab OR ‘older aged’:ti,ab OR ‘age related cognitive impairment*’:ti,ab OR ‘age-related cognitive impairment*’:ti,ab OR ‘ARCI’:ti,ab OR fragile:ti,ab OR fragility:ti,ab OR ‘aging adult*’:ti,ab OR ‘ageing adult*’:ti,ab) OR ('aged'/de OR 'frail elderly'/de OR 'very elderly'/de OR 'aging'/exp OR 'frailty'/de)

AND (((‘independent living’:ti,ab OR ‘living independently’:ti,ab OR ‘live independently’:ti,ab OR ‘lives independently’:ti,ab OR ‘community dwelling*’:ti,ab OR ‘community-dwelling*’:ti,ab OR ‘retirement communit*’:ti,ab OR ‘retirement cent*’:ti,ab OR ‘retirement life care cent*’:ti,ab OR ‘age in place’:ti,ab OR ‘aging in place’:ti,ab OR ‘ageing in place’:ti,ab OR ‘ages in place’:ti,ab OR ‘aged in place’:ti,ab OR ‘age at home’:ti,ab OR ‘aging at home’:ti,ab OR ‘ageing at home’:ti,ab OR ‘ages at home’:ti,ab OR ‘aged at home’:ti,ab OR ‘private residenc*’:ti,ab OR ‘domestic environment*’:ti,ab OR ‘home environment*’:ti,ab OR ‘reside at home’:ti,ab OR ‘resides at home’:ti,ab OR ‘residing at home’:ti,ab OR ‘resided at home’:ti,ab OR ‘assisted living’:ti,ab OR ‘living at home’:ti,ab OR ‘lives at home’:ti,ab OR ‘live at home’:ti,ab OR ‘lived at home’:ti,ab OR ‘community living’:ti,ab OR ‘congregate hous*’:ti,ab OR 'independent living'/de OR 'assisted living facility'/de OR 'community living'/de)

NOT ((protocol*:ti OR conference*:ti OR proceedings:ti OR ‘book review’:ti OR editorial:ti))

AND

#18 AND [2010-2021]/py AND [english]/lim AND ([article]/lim OR [article in press]/lim OR [data papers]/lim OR [erratum]/lim OR [review]/lim)
