# Supplementary material for: Is functional training functional? a systematic review of its effects in community-dwelling older adults
Source: Eur Rev Aging Phys Act. 2024 Dec 21;21:32. doi: 10.1186/s11556-024-00366-3 (PMC11664925; doi:10.1186/s11556-024-00366-3)
Supplement: Supplementary file 3 — Supplementary Material 3 [file 11556_2024_366_MOESM2_ESM.docx]

Appendix C.

Table 1. Summary table of reviewed studies (*n* = 32).

| **Study ID^a^**  **Country** | **Research Design**  **Characteristics of Study Participants** | **Characteristics of Intervention by Groups** | | **Outcome Measures**   - **ADLs/IADLs** - **Physical functioning** - **Cognitive function** | **Relevant Main Findings** |
| --- | --- | --- | --- | --- | --- |
|  |  | **Functional Training Group** | **Comparison Group** |  |  |
| **A. General community-dwelling older adults** | | | | | |
| **A.1. Single-component approach (*n* = 14)** | | | | | |
| Barcelona 2023 (44)  USA | RCT  Participants were all females and recruited from a local assisted living community.    Functional training group: n = 35; Mean age (SD) = 79.3 (0.9); Female n (%) = 35 (100); Drop-out n (%) = 2 (5.4) (Note that 35 out of 37 participants who completed the program were reported).    Active comparison group: n = 35; Mean age (SD) = 77.6 (1); Female n (%) = 35 (100); Drop-out n (%) = 2 (5.4) (Note that 35 out of 37 participants who completed the program were reported).  Control group: n = 20; Mean age (SD) = 78.5 (0.8); Female n (%) = 20 (100); Drop-out n (%) = 6 (23.1) (Note that 20 out of 26 participants who completed the program were reported). | *ADL exercise*   - A 5-minute warm-up, then a battery of ADL flexibility exercise, such as stepping into and out of the shower, and a 5-minute warm down. - One set of each ADL exercise activity in weeks 1-2, then two sets in weeks 3-4, and three sets in weeks 5-16. - Supervised by an occupational or physical therapist. - 16 weeks, 3 sessions per week. | *Active comparison group*   - Strength training in group. - 15 bilateral repetitions of 10 different strength exercises at 70% of 1-repetition maximun. - Supervised by an activity director. - Session progression, duration, and timeline are identical to the functional training group.   *Control group*  -Maintain usual activities. | *ADLs/IADLs.* None.  *Physical functioning.*   - Senior Fitness Test, which includes TUG, 30-second chair stand test, and 6-minute walk tests.   *Cognitive function.* None. | - The ADL exercise group was better than the control group on the TUG and 6-Minute Walk Test. - The strength training group was better than the ADL exercise group and the control group on the 30-second chair stand test, TUG, and 6-minute walk test.   ­­ |
| Burton 2013 (33)  Australia | Pragmatic RCT  Participants were recruited from a restorative home care service.  Functional training group:  n = 40; Mean age (SD) = 80.2 (6.4); Female *n* (%) = 30 (75); Drop-out *n* (%) = 1 (2.5).  Active comparison group:  n = 40; Mean age (SD) = 79.6 (6.2); Female *n* (%) = 36 (90); Drop-out *n* (%) = 3 (7.5). | *The LiFE Program*   - A trained care manager taught and provided a manual about the strategies to improve balance and increase strength and how to apply these strategies in everyday activities at home. - Every 10-14 days for 8 weeks, care manager checked in on clients and discussed the LiFE program (average 3 visits). | *Active control group*   - Structured exercise program which was based on the Otago falls prevention program. - Participants were given written handouts with illustrations of the exercise and the recommended schedule to complete the exercise. - 8 weeks. | *ADLs/IADLs.*   - LLFDI   *Physical functioning.*   - Functional reach test - Tandem walk - TUG. - 5 times chair stand test - One-time sit to stand test   *Cognitive function*. None. | - The LiFE group showed a significant improvement in the limitation subscale, instrumental role, and management role of the LLFDI than the structured exercise group. No group differences were found in other LLFDI subscales. - Mixed results in the functional balance between the functional training group and active comparison group (significant difference in tandem walk but not in the functional reach test). - No group differences in functional lower body strength and mobility. |
| Clemson 2012 (29)  Australia | RCT  Participants were recruited from two registered databases.  Functional training group:  *n* = 107; Mean age (SD) = 82.8 (4.2); Female *n* (%) = 59 (55.1); Drop-out *n* (%) = 19 (17.8).  Active comparison group:  *n* = 105; Mean age (SD) = 84 (4.4); Female *n* (%) = 57 (54.3); Drop-out *n* (%) = 22 (21).  Sham control group:  *n* = 105; Mean age (SD) = 83.5 (3.8); Female *n* (%) = 58 (55.2); Drop-out *n* (%) = 19 (18.1). | *LiFE program*   - Teaching strategies to improve balance and increase strength and applying these strategies in everyday activities. - The program was home-based and taught over five group sessions (60-90 minutes per session, one session per week) with two booster sessions and two follow-up phone calls over six months. | *Active comparison group*   - Structured balance and lower limb strength exercises in three sessions per week. - The program was taught over five sessions with two booster sessions and two follow-up phone calls over six months.   *Sham control group*   - Gentle and flexibility seated exercises. - Two sessions, one booster session and six follow-up phone calls. | *ADLs/IADLs.*   - LLFDI. - National Health and Nutrition Examination Survey (NHANES) ADL.   *Physical functioning.*   - Five-level static balance scale. - Eight-level static balance scale. - 3-meter tandem walk test (dynamic balance).   *Cognitive function.* None. | - Compared with the sham control group, the LiFE group showed a larger effect size than the structured exercise group on ADL/IADL performance. - Compared with the sham control group, both the LiFE and structured exercise groups significantly improved in static and dynamic balance. The LiFE group showed a larger effect size in static balance than the structured exercise group. |
| Hu 2023 (43)  USA | RCT  Medically underserved participants were recruited locally or from a previous study.  Functional training group: n = 8; Mean age (SD) = 76 (4.9); Female n (%) = 6 (75); Drop-out n (%) = 0 (0).  Active comparison group: n = 8; Mean age (SD) = 78.5 (6.9); Female n (%) = 6 (75); Drop-out n (%) = 0 (0). | *DO LiFE (modified LiFE program)*   - Balance and muscle strength exercises were embedded in daily routine activities via habit formation strategies. - Delivered by an occupational therapist in the participant’s home. - 7 sessions, 20-60 minutes per session. - First 5 sessions weekly basis, 6^th^ session 2 week after 5^th^, and 7^th^ session one month after 6^th^. | *Active comparison group*   - Go4LiFE 19 flexibility exercise activities. - Delivered by an occupational therapist in the participant’s home. - Session formation, duration, and timeline are identical to the functional training group. | *ADLs/IADLs.* None.  *Physical functioning.*   - Berg Balance Scale. - Short Physical Performance Battery.   *Cognitive function.* None. | - No group differences were found in physical functioning measures. |
| Hummer 2015 (34)  USA | Singe group pre-post test design  Participants were recruited from an assisted living facility.  Functional training group:  *n* = 25; Median age (Range) = 88.5 (71-97); Female *n* (%) = 20 (80); Drop-out *n* (%) = 3 (12). | *Sit-to-Stand Exercise Program*   - Perform up to 5 sit-to-stand exercises during each session. - Delivered by certified nursing assistants near the common dining area. - 90 days, every time before lunch and dinner. | No comparison group. | *ADLs/IADLs.*   - Katz ADL Index.   *Physical functioning.* None.  *Cognitive function.* None. | - No statistical significance was identified in ADL performance. - Note that the implementation was limited given that participants attended a median of 13% of the planned sessions. |
| Karóczi 2014 (41)  Hungary | Quasi-RCT  Participants were recruited locally through newsletters.  Functional training group:  *n* = 18; Mean age (SD) = 71.5 (6.2); Female *n* (%) = 3 (16.7); Drop-out *n* (%) = 3 (16.7).  Control group  *n* = 18; Mean age (SD) = 70.6 (5.1); Female *n* (%) = 3 (16.7); Drop-out *n* (%) = 0 (0). | *Group-based functional balance training program*   - Exercises simulating everyday activities (i.e., sit-to-stand, sit down, turning, squatting, reaching in different directions, stepping up and down). - First 5 to 6 weeks: Exercises were practiced separately - After 5 to 6 weeks: Exercises were practiced combined with additional tasks (i.e., holding and carrying glass or tray). - Delivered by a physical therapist and physical therapy student at a local sports center. - 25 weeks, 60 min per session, two sessions a week. | *Control group*  Usual daily activities. | *ADLs/IADLs.* None.  *Physical functioning.*   - Fullerton test. - TUG. - Five-times sit to stand test. - Two-minute-step-in-place test.   *Cognitive function.* None. | - Significant improvement in the functional training group on static and dynamic balance (Fullerton test) and mobility (TUG) compared to the control group. No group differences were found in other physical functioning tests. |
| King 2012 (35)  New Zealand | Cluster RCT  Older people were recruited from a single home care agency.  Functional training group:  *n* = 93; Mean age (SD) = 80.5 (6.3); Female *n* (%) = 72 (77.4); Drop-out *n* (%) after intervention = 11 (11.8), after follow-up = 17 (18.3).  Control group:  *n* = 93; Mean age (SD) = 78.4 (6.5); Female *n* (%) = 65 (69.9); Drop-out *n* (%) after intervention = 11 (11.8), after follow-up = 12 (12.9). | *Enhanced training and supervision for paid caregivers, care management, goal facilitation, and a restorative care philosophy. The multifaceted approach included repetitive ADL exercises*   - Specific functional ADL exercises (including lifting a grocery bag, containing cans, and carrying) based on individualized goals. - The exercise was supervised by paid caregivers at the participant’s home. - The intervention dose was not specified. | *Control group*   - The paid caregivers supported household activities or personal care. | *ADLs/IADLs.*   - Nottingham Extended ADL Scale.   *Physical functioning.*   - TUG. - SF36 Physical.   *Cognitive function*. None. | - No significant group differences were found in functional mobility, overall physical performance, and ADL/IADL measures. |
| Li 2018 (42)  USA | Single group pre-post test design  Participants were recruited from retirement communities.  Functional training group:  *n* = 16; Mean age (SD) = 87.9 (4.8); Female *n* (%) = 14 (87.5); Drop-out *n* (%) = 3 (18.8) (Note that 16 out of 19 participants completed the program and their data were reported). | *Modified version of LiFE program*   - Lower limb muscle strengthening and balance exercises were integrated into everyday activities via discussion and simulated demonstrations. The program started with five group sessions in the community activity room in the first five weeks. One Booster session was provided in Week 10. Two phone call follow-ups were provided between Week 10 and 26. - The group sessions were provided by occupational therapy students, from Week 2 to 6, 60-90 min per session, 1 session per week. | No comparison group. | *ADLs/IADLs.* None.  *Physical functioning.*   - 30-second chair stand test. - TUG. - One-legged Stand. - Functional reach test.   *Cognitive function.* None. | - All physical functioning performance tests, except the one-legged Stand, were improved from pretest to posttest and pretest to follow-up. |
| Liu 2016 (38)  USA | Single group pre-post test design  Participants were recruited from two subsidized senior housing communities.  Functional training group:  *n* = 14; Mean age (SD) = 73.3 (6.8); Female *n* (%) = 11 (78.6); Drop-out *n* (%) = 3 (17.7) (Note that 14 out of 17 participants who completed the program were reported). | *3-Step Workout for Life program*   - Started with group-based progressive resistance training, and then added one-on-one functional exercise and individualized ADL exercise. The three exercises were delivered in a stepwise manner. - Exercise intensity was kept at moderate intensity. - Delivered by train research personnel at participants’ community and home. - 10 weeks, 50-60 min per session, three sessions a week. | - No comparison group. | *ADLs/IADLs.*   - LLFDI. - AMPS Motor Skills.   *Physical functioning*   - Box and Block Test. - 30 seconds chair stand test.   *Cognitive function.* None. | - After the intervention, gross hand function (Box and Block Test) and functional lower body strength (30 seconds chair stand test), subjective ADL/IADL performance (LLFDI-function), and objective ADL/IADL performance (AMPS Motor Skills) were significantly improved. |
| Liu 2017 (39)  USA | RCT  Participants were recruited from local subsidized senior housing communities.  Functional training group:  *n* = 27; Mean age (SD) = 71.7 (8.2); Female *n* (%) = 21 (77.8); Drop-out *n* (%) = 6 (22.2).  Active comparison group:  *n* = 25; Mean age (SD) = 73.6 (7.7); Female *n* (%) = 22 (88); Drop-out *n* (%) = 2 (8). | *3-Step Workout for Life program*   - Started with group-based progressive resistance training, and then added one-on-one functional exercise and individualized ADL exercise. The three exercises were delivered in a stepwise manner. - Delivered by trained research personnel at participants’ community and home,10 weeks, 60 minutes per session, and three sessions a week. | *Active comparison group*   - Resistance Exercise on major muscle groups of the upper and lower extremities. - 10 weeks, 60 minutes per session, and three sessions a week. | *ADLs/IADLs.*   - AMPS Motor Skills.   *Physical functioning.*   - Box and Blocks Test. - TUG.   *Cognitive function*. None. | - While there was no significant difference between groups in most measurements at post-intervention and 6-month follow-up, the functional training group showed a significantly greater performance in the AMPS Motor Skills at the 6-month follow-up. |
| Liu 2020 (30)  USA | Singe group pre-post test design  Participants were recruited from a local retirement community.  Functional training group:  *n* = 13; Mean age (SD) = 81.23 (8.7); Female *n* (%) = 8 (61.5); Drop-out *n* (%) = 4 (23.5) (Note that 13 out of 17 participants who completed the program were reported). | *3-Step Workout for Life program*   - Started with group-based progressive resistance training, and then added group-based functional exercise and individualized ADL exercise. The three exercises were delivered in a stepwise manner. - Exercise intensity was kept at moderate intensity. - Delivered by community fitness coordinators at the community gym and participants’ home. - 10 weeks, 50-60 min per session, three sessions a week. | - No comparison group. | *ADLs/IADLs.*   - AMPS Motor Skills. - Canadian Occupational Performance Measure.   *Physical functioning.*   - 30-second chair stand test. - Box and Blocks Test. - TUG.   *Cognitive function.* None. | - Participants significantly improved in all outcome measures, except the 30-second chair stand test. |
| Mohammed 2022 (36)  India | RCT  Participants were recruited from recreational parks and religious places.  Functional training group:  *n* = 50; Mean age (SD) = 73.4 (4.2); Female *n* (%) = 21 (42); Drop-out *n* (%) = 4 (8).  Active comparison group:  *n* = 50; Mean age (SD) = 72.8 (4.2); Female *n* (%) = 19 (38); Drop-out *n* (%) = 9 (18). | *Functional Task Training*   - 10-minute warm-up of aerobic exercise, 40-minute of functional task training program, and 10-minute cool-down of flexibility exercise. - Functional task training involves exercises that are similar to daily activities such as picking up an object from the floor and placing it on a shelf. The exercises were progressively increased in complexity and variability. - The training was in a physical therapy unit of a local hospital. - 12 weeks, 60 minutes per session, and three sessions a week. | *Active comparison group*   - 10-minute warm-up of aerobic exercise, 40-minute of progressive resistance exercise, and 10-minute cool-down of flexibility exercise. - Progressive resistance exercise includes four resistance exercises. - The exercise duration, frequency, and location were identical to the other group. | *ADLs/IADLs.*   - Groningen Activity Restriction Scale.   *Physical functioning.* None.  *Cognitive function.* None. | - The improvement of IADL/ADL performance was significantly greater in the functional task training group than in the resistance exercise group. |
| Siemonsma 2018 (37)  Netherlands | RCT  Participants with health problems were recruited from primary care practices.  Functional training group:  *n* = 76; Mean age = 84 (25 percentile = 79.4), 75 percentile = 88.7); Female *n* (%) = 55 (72); Drop-out *n* (%) = 27 (35.5).  Active comparison group:  *n* = 79; Mean age = 83.9 (25 percentile = 80.2, 75 percentile = 86.4); Female *n* (%) = 59 (75); Drop-out *n* (%) = 20 (25.3).  Control group  *n* = 228; Mean age = 84.7 (25 percentile = 80.5, 75 percentile = 89.5); Female *n* (%) = 179 (79); Drop-out *n* (%) = 81 (35.5). | *Functional task exercise*   - Problem-oriented, situational, and task-specific training provided by physical therapists. - Activities were trained by exercises that closely linked cognition, perception, and execution of tasks in relation to participants’ home environment. - Participants’ social environment was engaged. - Participants were monitored and exercise intensity was adjusted to achieve reserves in both physical, perceptual, and cognitive capacities. - 3 months, 30 min per session, maximum 18 individual sessions. | *Active comparison group*   - Preventive physical therapy in the clinic or at home. - Any exercises therapy and advice were up to the therapist’s discretion. - Followed the Royal Dutch Society for Physical Therapy protocols. - 3 months, 30 min per session, maximum 18 individual sessions.   *Control group*   - Functioning data from an observational study. | *ADLs/IADLs.*   - Modified Katz-15. - Groningen Activity Restriction Scale.   *Physical functioning.* None.  *Cognitive function.* None. | - No statistically significant results were observed between the functional task exercise group and the preventive physical therapy group. - Deterioration in intervention trial groups (functional task exercise group and preventive physical therapy group) was significantly less in both ADL/IADL measures compared to the control group. |
| Solberg 2013 (40)  Norway | RCT  Participants were recruited through advertisements and word of mouth.  Functional training group:  *n* = 33; Mean age (SD) = 73.4 (3.7); Female *n* (%) = 15 (60); Drop-out *n* (%) = 8 (24.2).  Active comparison group 1:  *n* = 33; Mean age (SD) = 75 (6); Female *n* (%) = 20 (67); Drop-out *n* (%) = 3 (9.1).  Active comparison group 2:  *n* = 33; Mean age (SD) = 73.9 (3.7); Female *n* (%) = 21 (70); Drop-out *n* (%) = 3 (9.1).  Control group:  *n* = 39; Mean age (SD) = 74.6 (4.5); Female *n* (%) = 24 (73); Drop-out *n* (%) = 6 (15.4). | *Functional strength training*   - Consisted of exercise movements that mimicked ADLs (three leg and four upper body exercises such as rise from chair and case lift). - 13 weeks, about 60 min per session, 3 sessions a week. | *Active comparison group 1*   - Traditional strength training on major muscle groups.   *Active comparison group 2*   - Endurance Training using Nordic walking, aerobics, and hiking on rugged terrain.   Both active comparison groups received training for 13 weeks, 60 minutes each session for 3 sessions a week.  *Control group*   - Wait-list. | *ADLs/IADLs. None.*  *Physical functioning.*   - Stair climbing without load and with load. - TUG. - Five chair stand test. - Functional upper body strength test. - Six-minute walk test. - 10-meter walking at normal and fast speed.   *Cognitive function*. None. | - Functional strength training group showed significant improvement in the loaded stair climbing test more than the control group. - All groups increased their performance in the functional upper body strength test, but the improvement was larger in the functional training group and strength training group than in the endurance training and control groups. - No group differences were found in the three walk tests and the chair stand test. |
| **A2. Multi-component approach (*n* = 4)** | | | | | |
| Comans 2010 (45)  Australia | RCT  Participants were recruited from a regional community rehabilitation service.  Functional training group:  *n* = 55; Mean age (SD) = 78.7 (8); Female *n* (%) = 37 (67.3); Drop-out *n* (%) = 14 (25.5).  Active comparison group:  *n* = 52; Mean age (SD) = 79.2 (7.4); Female *n* (%) = 34 (65.4); Drop-out *n* (%) = 18 (34.6). | *Domiciliary Program (home-based program)*   - The program included balance and strength exercises, education on fall prevention, functional task exercise, and home modification recommendations. The functional task exercise may include cooking and hanging out washing. - 8-week, 60 minutes per session, one session delivered by a therapist per week at the participant’s home. - Home exercise program: Performed twice daily for around 10 minutes at each session. - Fall prevention education: 30 minutes of verbal presentation with handout. | *Active comparison group (Center-based Program)*   - The exercise and education modules were same as the Domiciliary exercise program. - The functional tasks module included upper limb strengthening and functional activities (e.g., shoulder arc, pegboard) in standing. - 8 weeks, 60 minutes per session, one session per week delivered by a therapist at a hospital gym. | *ADLs/IADLs.*   - Frenchay Activities Index.   *Physical functioning.*   - 9-hole peg test. - Step test. - TUG.   *Cognitive function.*   - Abbreviated mental test | - No group differences were found in all measures except that the center-based group performed better on the upper limb dexterity than the Domiciliary group. |
| Oh 2021 (47)  South Korea | RCT  Participants were recruited from a senior housing apartment.  Functional training group 1 (task-oriented + motor imagery):  *n* = 11; Mean age (SD) = 79.9 (5.6); Female *n* (%) = 7 (63.6); Drop-out *n* (%) = 1 (8.3). Note that 11 out of 12 participants completed the study and were analyzed.  Functional training group 2 (task-oriented only):  *n* = 11; Mean age (SD) = 78.7 (2.6); Female *n* (%) = 8 (72.7); Drop-out *n* (%) = 1 (8.3). Note that 11 out of 12 participants completed the study and were analyzed.  Control group:  *n* = 12; Mean age (SD) = 77.2 (2.9); Female *n* (%) = 8 (66.7); Drop-out *n* (%) = 0 (0). | *Task-oriented training + motor imagery training group (functional training group 1)*   - The task-oriented training entails balance training centered on daily activities in a real-life environment. For example, putting a cup on the shelf or replacing bathroom towels. - The motor imagery training asks the participants to picture protective body movements in the event of falls. For example, losing balance at the moment of getting out of bed at night and walking through the dark room. - Trainings were performed at a senior citizen center. - 6 weeks, 40 min per session, 18 sessions.   *Task-oriented training only group (functional training group 2)*   - Only received task-oriented training as described above. - 6 weeks, 40 min, 18 sessions. | *Control group*   - Educated on fall prevention and health using audio-visual materials. - 6 weeks, 40 min, 18 sessions. | *ADLs/IADLs.* None.  *Physical functioning.*   - Berg Balance Scale. - TUG. - Gait evaluation using the GAITRite system.   *Cognitive function.* None. | - Both groups that applied task-oriented training significantly improved on mobility, and static and dynamic balance at the posttest compared to the control group. - The improvement was significantly larger in the combined task-oriented training and motor imagery training group than in the task-oriented training only group on mobility, static, and dynamic balance. - No difference was found in the gait evaluation between the two intervention groups. |
| Szanton 2021 (46)  USA | RCT  Participants were recruited from senior housing and prior studies.  Functional training group:  *n* = 25; Mean age (SD) = 78.5 (7.3); Female *n* (%) = 15 (60); Drop-out *n* (%) = 2 (8).  Control group:  *n* = 12; Mean age (SD) = 76.5 (3.6); Female *n* (%) = 9 (75); Drop-out *n* (%) = 0 (0). | *The LIVE-LiFE intervention*   - Four components: The LiFE approach plus home safety changes, vision contrast screening and referral, and medication recommendations. - The intervention was provided by an occupational therapist at the participant’s home. - The LiFE approach involved teaching strategies to improve balance and increase strength and applying these strategies in everyday activities. - One-hour home visit sessions once a week from Week 1 to 6; One-hour home visit booster sessions once a week from Week 8 and 12. 60 minutes per session. | *Control group*   - Received a letter with their scores on the fall risk screening assessment to bring to their primary care providers. - Received a CDC pamphlet about fall prevention which included steps to take to reduce fall risk. | *ADLs/IADLs.* None.  *Physical functioning.*   - TUG. - Tandem stand test.   *Cognitive function.* None. | - LIVE-LiFE group showed a significantly greater improvement in balance compared to the control group, while there was no group difference in mobility (TUG). |
| Todo 2021 (31)  Japan | Singe group pre-post test design  Participants were recruited from a home-based rehabilitation service facility.  Functional training group:  *n* = 35; Mean age (SD) = 82.4 (7.5); Female *n* (%) = 24 (80.0); Drop-out *n* (%) = 5 (14.3). Note that 30 out of 35 participants. completed the study and were analyzed. | *Multicomponent Home-Based Rehabilitation Program*   - Multi-modal exercise included stretching, range of motion, strengthening, and balance exercises. - Practicing ADLs and techniques, e.g., bed mobility, toileting, meal preparation, dish washing. - Improving home environment: Removing environmental hazards, providing assistive devices, and modifying the home. - Caregiver support: Guidance regarding safer techniques for assisting participant ADLs, fall-recovery techniques, using assistive devices, and removing environmental hazards. - The program was delivered by physical and occupational therapists at the participant’s home. - 3 months, 40-60 min per visit, at least once or twice a week. | No comparison group | *ADLs/IADLs.*   - Functional Independence Measure.   *Physical functioning.*   - Ability to stand without assistance.   *Cognitive function.* None. | - ADL performance and the number of participants who were able to stand without assistance were improved significantly after the intervention. |
| **A 3. The dual-task paradigm approach (*n* = 2)** | | | | | |
| Halvarsson 2011 (32)  Sweden | RCT  Participants were recruited by newspaper advertisements.  Functional training group:  *n* = 38; Mean age (range) = 76 (67-93); Female *n* (%) = 25 (65.8); Drop-out *n* (%) = 4 (10.5).  Control group:  *n* = 21; Mean age (range) = 78 (69-91); Female *n* (%) = 17 (81); Drop-out *n* (%) = 0 (0). | *Individually Adjusted, Progressive and Specific Balance Group Training*   - Dual or multi-cognitive and/or motor task (e.g., counting, reading a newspaper, doing up/undoing buttons or carrying a tray with glasses of water while walking forwards, backwards, or around cones) at five levels. - 12 weeks, 45 min per session, three sessions a week. | *Control group*   - To continue life as usual. | *ADLs/IADLs.* None.  *Physical functioning.*   - Gait evaluation using the GAITRite system.   *Cognitive function.* None. | - The intervention group improved more in the gait velocity than the control group. No group differences in other gait evaluation outcomes. |
| Nott 2019 (48)  Australia | Two-group, non-randomized, waitlist controlled study design.  Participants were recruited through media invitations and posters at local community centers.  Functional training group:  *n* = 16; Mean (SD) = 74 (8); Female *n* (%) = 10 (62.5); Drop-out *n* (%) = 5 (31.3).  Control group:  *n* = 12; Mean age (SD) = 77 (6); Female *n* (%) = 11 (91.7); Drop-out *n* (%) = 0 (0). | *Aging well program*   - Activities were designed to challenge motor and cognitive abilities (dual tasks) and graded from simple to complex. - Activities simulated daily functional tasks or used skills integral to daily functional tasks such as balance, mobility and strength, planning, problem-solving, attention, and memory. For example, the ADL obstacle course. - Activities were performed in a range of contexts, such as nearby café and supermarkets. - Each session was facilitated by at least three allied health professionals and up to eight health students. - 10 weeks, 60-90 min per session, one session per week. | *Control group*   - On the waitlist. | *ADLs/IADLs.* None.  *Physical functioning.*   - TUG. - Six Minute Walk Test.   *Cognitive function.*   - MMSE | - No difference was found in lower body function, mobility, and global cognition between the two groups. |
| **B. Community-dwelling older adults with cognitive impairment** | | | | | |
| **B1. Older adults with mild cognitive impairment: Single-component approach (*n* = 4)** | | | | | |
| de Freitas 2021 (49)  Australia | Single group, pre-post test design  Participants with subjective cognitive concerns and objective cognitive impairment were recruited via word of mouth amongst community groups.  Functional training group:  *n* = 23; Age range 60~70 *n* = 6, 71~80 *n* = 8, 81~90 *n* = 7, and 91~100 *n* = 2; Female *n* (%) = 19 (82.6); Drop-out *n* (%) = 3 (13). | *Simulated functional tasks exercise (FcTSim)*   - Combined exercise and cognitive retraining. Participants performed a chair raise movement as the exercise component while using unilateral and bimanual movements, task switching and/or body midline crossing in a prespecified sequence as the cognitive component. The prespecified sequence was the placing and collection of bowls and cups to mimic everyday living in a specific pattern. - Facilitated by an occupational therapy assistant and supervised by an occupational therapist. - Mixed with center-based and home-based training sessions. - 10 weeks, 40-60 min per session (30-40 min for exercise), three sessions a week. | No comparison group. | *ADLs/IADLs.*   - Lawton IADL scale.   *Physical functioning.* None.  *Cognitive function.*   - NCSE. - PEDL. - TMT. - VFT. - VLT. | - IADL performance, general cognitive function, executive function, delayed recall, and everyday problem-solving ability significantly improved after the intervention and sustained at 3-month follow-up. No difference was found in executive function. |
| Law 2013 (18)  Hong Kong | Single-group, pre-post test design  Participants with subjective memory complaints or suspected cognitive impairment were recruited from an outpatient clinic.  Functional training group:  *n* = 11; Mean age (SD) = 71.82 (8.5); Female *n* (%) = 9 (81.8); Drop-out *n* (%) = 0 (0). | *Simulated functional tasks exercise (FcTSim)*   - Placing two different sets of utensils (cups and bowls) following rules and specific movement patterns. - Five different levels of task components. - Perform sit-to-stand exercise between the specific movement patterns (unilateral, bimanual movement, task switching, and body midline crossing). - The program was facilitated by an occupational therapist. - Mixed with center-based and home-based training sessions. - 10 weeks, 40-60 min per session (30-40 min for exercise), and 3 sessions per week. | No comparison group. | *ADLs/IADLs.*   - Lawton IADL scale.   *Physical functioning.* None.  *Cognitive function.*   - CVVLT. - NCSE. - PEDL. - VFT. | - Significant improvement in all interested outcomes and the effects were sustained at the 6-month follow-up. |
| Law 2014 (17)  Hong Kong | RCT  Patients with a subjective memory complaint or suspected cognitive impairment were referred to the study by a medical department.  Functional training group:  *n* = 43; Mean age (SD) = 73.69 (6.8); Female *n* (%) = 27 (62.8); Drop-out *n* (%) = 2 (4.7).  Active comparison group:  *n* = 40; Mean age (SD) = 74.1 (7.6); Female *n* (%) = 23 (57.5); Drop-out *n* (%) = 6 (15). | *Simulated functional tasks exercise (FcTSim)*   - Placing two different sets of utensils (cups and bowls) following rules and specific movement patterns. - Five different levels of task components. - Perform sit-to-stand exercise between the specific movement patterns (unilateral, bimanual movement, task switching, and body midline crossing). - The program was facilitated by an occupational therapist. - Mixed with center-based and home-based training sessions. - 10 weeks, 40-50 min per session (30 min for exercise), total 13 sessions. | *Active comparison group*   - Cognitive training program consisted of 30 minutes of computer-based cognitive training and 30 minutes of cognitive strategy training. - Facilitated by an occupational therapist and an assistant. - 10 weeks, 60 min per session, 6 sessions. | *ADLs/IADLs.*   - Lawton IADL scale.   *Physical functioning.* None.  *Cognitive function.*   - CVFT. - CVVLT. - NCSE. - PEDL. - TMT. | - The FcTSim group showed a significant improvement in IADL performance, general cognitive functions, immediate and delayed recall, executive function, and everyday problem-solving ability compared to the cognitive training group. - These differences in general cognitive functions, memory, executive function, and everyday problem-solving ability were maintained in the 6-month follow-up. |
| Law 2018 (19)  Hong Kong | Single-group, pre-post test design.  Patients with subjective memory/cognitive complaints or objective cognitive impairment revealed by neuropsychological assessment were recruited from an outpatient clinic.  Functional training group:  *n* = 43; Mean age (SD) = 73.56 (6.8); Female *n* (%) = 27 (62.8); Drop-out *n* (%) = 1 (2.3). | *Simulated functional tasks exercise (FcTSim)*   - Involving the performance of simulated functional tasks (cup/bowl placing and collection) following specific patterns of movement and sequence incorporated with a sit-stand movement performed after placing each cup/bowl. - Activity speed was progressed to maintained at a moderate intensity level. - Delivered by an occupational therapist. - Mixed with center-based and home-based training sessions. - 10 weeks, 40-50 min per session (30 min for exercise), total 13 sessions. | No comparison group. | *ADLs/IADLs.*   - ADL Questionnaire.   *Physical functioning.*   - 30-second chair stand test.   *Cognitive function.*   - CVFT. - NCSE. - PEDL. - TMT. | - Significant improvement in all interested outcomes at intervention completion. |
| **B2. Older adults with mild cognitive impairment: Multi-component approach (*n* = 1)** | | | | | |
| Liao 2020 (50)  Taiwan | RCT  Participants were recruited from communities and daycare centers.  Functional training group:  *n* = 18; Mean age (SD) = 75.5 (5.2); Female *n* (%) = 11 (61.1); Drop-out *n* (%) = 3 (16.7).  Active comparison group:  *n* = 16; Mean age (SD) = 73.1 (6.8); Female *n* (%) = 12 (75); Drop-out *n* (%) = 5 (31.3). | *VR-Based physical and cognitive training*   - VR-based physical training included multi-modal exercise and functional tasks, such as window cleaning and goldfish scooping conducted by a physical therapist. - VR-based cognitive training was games based on actual IADLs, such as shopping, food preparation, and the use of public transportation. - 12 weeks, 60 min per session (40 min for VR-based exercises and 20 min for cognitive training), three sessions a week. | *Active comparison group*   - Combined multimodal exercise and cognitive training simultaneously. - The multimodal exercise included resistance, aerobic, balance, and mobility exercises. - Cognition training (e.g., reciting poems, and calculations) was delivered concurrently with exercise training. - 12 weeks, 60 min per session, three sessions a week | *ADLs/IADLs.*   - Lawton IADL scale.   *Physical functioning.* None.  *Cognitive function.*   - CVVLT. - Montreal Cognitive Assessment. - The Executive Interview 25. | - The IADL performance is the only outcome showing the superior effect of VR-based functional training. |
| **B3. Community-dwelling older adults with dementia: Single-component approach (*n* = 2)** | | | | | |
| Hauer 2012 (51)  Germany | RCT  Participants with confirmed mild to moderate dementia were recruited from rehabilitation wards of a geriatric hospital.  Functional training group:  *n* = 62; Mean age (SD) = 82.3 (6.6); Female *n* (%) = 46 (74.2); Drop-out *n* (%) = 14 (22.6).  Active comparison group:  *n* = 60; Mean age (SD) = 82.9 (7); Female *n* (%) = 44 (73.3); Drop-out *n* (%) = 9 (15). | *Progressive resistance and functional training.*   - Combined progressive resistance and functional training supervised by a qualified instructor. - Resistance training focused on functionally relevant muscle groups at submaximal intensity in groups of four to six participants. - Functional training included performing basic ADL-related motor functions such as chair stand, and stair climb with various levels. - 3 months, 2 hours per session, twice a week. | *Active comparison group*   - Low-intensity multimodal exercise. - 3 months, 1 hour per session, two sessions a week. | *ADLs/IADLs.* None.  *Physical functioning.*   - Five chair stands and stair climbing from the Modified Short Physical Performance Battery. - Performance Oriented Motor Assessment (balance and mobility). - TUG.   *Cognitive function.* None. | - The intervention group showed better outcomes in functional lower body strength, mobility, and static and dynamic balance than the comparison group at training completion and the 3-month follow-up. |
| Pedroso 2018 (52)  Brazil | Quasi-group design  Participants with a clinical diagnosis of Alzheimer’s disease were recruited from an existing program.  Functional training group:  n = 22; Mean age (SD) = 77.6 (6.2); Female *n* (%) = 16 (72.7); Drop-out *n* (%) = 6 (21.4) (only 22 participants among 28 who completed the program were analyzed).  Social gathering group:  n = 21; Mean age (SD) = 78 (5.6); Female *n* (%) = 16 (76.2); Drop-out *n* (%) = 4 (16) (only 21 participants among 25 who completed the program were analyzed).  Control group  n = 14; Mean age (SD) = 79.2 (5.6); Female *n* (%) = 12 (85.7); Drop-out *n* (%) = 0 (0). | *Functional-task training*   - Simulated locomotion activities (e.g., Hanging out the washing, walking and carrying objects). - ADL stimulation activities (e.g., imitating dying one’s back with a towel). - Increases complexity and variability of the exercises in three phases: 2, 4, and 6 weeks in duration. - 12 weeks, 60 min per session, three sessions a week. | *Active comparison group*  Social gathering group   - Included group activities (e.g., singing and dancing), short walks, and recreational activities (e.g., watching movies). - Minimum of 2 activities per session. - Delivered by psychologists, gerontologists, and physical education professionals. - 12 weeks, 60 min per session, three sessions a week.   *Control group*   - Followed individual’s standard medical care. | *ADLs/IADLs.*   - Revised Direct Assessment of Functional Status.   *Physical* functioning.   - Senior Fitness Test Battery. - Tinetti Balance Scale.   *Cognitive function.*   - Clinical Dementia Rating. - Digit Span test. - MMSE. - TMT. - Toulouse-Pieron Concentrated Attention Test. - VFT. | - No significant group differences in ADL performance, balance, mobility, and cognitive functions. The arm curl result from the Senior Fitness Test is the only outcome showing the superior effect of the functional-task training compared to the control group. |
| **B4. Older adults with mild cognitive impairment or dementia: Multi-component approach (*n* = 2)** | | | | | |
| Harwood 2023 (54)  United Kingdom | RCT  Participants were recruited from secondary care memory clinics, general practice registers, dementia support groups and national register.  Functional training group:  *n* = 183; Median age = 80; Female *n* (%) = 82 (44.8); Drop-out *n* (%) = 34  (18.6).  Active comparison group:  *n* = 182; Median age = 81; Female *n* (%) = 73 (40.1); Drop-out *n* (%) = 41 (22.5). | *Promoting Activity, Independence, and Stability in Early Dementia and Mild Cognitive Impairment (PrAISED)*   - Individually tailored program of physical exercises, functional activities, inclusion in community life, risk enablement, and environmental assessment. - Up to 50 home therapy sessions over 12 months from a multidisciplinary team of occupational therapists, physical therapists, and rehabilitation support workers. - The frequency tapered down from two visits weekly to one visit monthly. | *Active comparison group*   - Falls prevention assessment and three advice visits - Provided by the same therapists in the intervention group | *ADLs/IADLs.*   - Disability assessment for dementia. - Nottingham Extended ADL Scale.   *Physical* functioning.   - Berg balance scale - TUG (single- and dual-task)   *Cognitive function.*   - Montreal cognitive assessment scale - Verbal fluency test - Cambridge neuropsychological test automated battery | - No group difference in the disability measure and ADL scale. - No group difference in the Berg balance scale and TUG, but the control group showed a better outcome on the dual task TUG. - The PrAISED group showed better results in executive function and visual-spatial working memory, as measured by the Cambridge neuropsychological test automated battery. |
| Straubmeier 2017 (53)  Germany | Cluster RCT  Participants were from daycare centers.  Functional training group:  *n* = 208; Mean age (SD) = 81.5 (7.5); Female *n* (%) = 127 (61.1); Drop-out *n* (%) = 55 (20.9).  Control group:  *n* = 154; Mean age (SD) = 81.1 (7.5); Female *n* (%) = 94 (61); Drop-out *n* (%) = 36 (19). | *Multicomponent therapy (Motor, activities of daily living, cognitive, social)*   - Social warm-up, such as a greeting round (10 min). - Sensorimotor activation (30 min), including general mobility, gross and fine motor skills, balance, and sensory perception. - Cognitive activation (30 min), including memorizing, recognizing, and forming associations using projector or pen-and-paper exercises. - Activation of activities of daily living (40 min), including housework or craftwork. - Trainings were performed at the daycare center. - 6 months, about 2 hours per session, every weekday. | *Control group*   - Usual care in daycare center. | *ADLs/IADLs.*   - Nottingham Extended ADL Scale.   *Physical functioning.* None.  *Cognitive function.*   - MMSE. | - The multicomponent group maintained ADL performance and global cognition while the control group deteriorated. |
| **C. Frail older adults** | | | | | |
| **C1. Single-component approach (*n* = 2)** | | | | | |
| Faria 2023 (56)  Portugal | RCT  Participants who had enrolled in a health care unit were recruited.  Functional training group: *n* = 15; Mean age (SD) = 80.07 (4.9); Female *n* (%) = 11 (73); Drop-out *n* (%) = 0 (0).  Control group:  *n* = 15; Mean age (SD) = 81.8 (5); Female *n* (%) = 10 (67); Drop-out *n* (%) = 0 (0). | *Active Aging-in-Place–Rehabilitation Nursing Program*   - Structured and individualized multicomponent programs that combine strength training, endurance, balance, flexibility, and ADL training. - ADL training is the simulation of functional movements that enable daily activities, such as bathing and grooming. - The exercises were adapted to each participant in each session. - Empowerment strategies to promote motivation and adherence. - 12 weeks, 24 sessions, 60 min per session. | *Control group*   - Maintain normal activities. | *ADLs/IADLs.*   - Barthel Index. - The Lawton IADL scale.   *Physical functioning.*   - Senior Fitness Test: 5 subtests, including TUG and chair stand test. - Tinetti Balance Index   *Cognitive function.* None. | - Results of pre-post tests in each group were reported. No direct group comparisons. - The functional training group improved in ADLs and all physical functioning measures from baseline to post-program but showed no improvement in IADLs. - The control group only improved in functional mobility and balance. |
| Oosting 2012 (55)  Netherlands | RCT  Participants with osteoarthritis and ready for total hip arthroplasty were recruited from an orthopedic department.  Functional training group:  *n* = 15; Mean age (SD) = 76.9 (6.3); Female *n* (%) = 14 (93); Drop-out *n* (%) = 0 (0).  Control group:  *n* = 15; Mean age (SD) = 75.0 (6.3); Female *n* (%) = 10 (67); Drop-out *n* (%) = 3 (20). | *Multicomponent home-based preoperative training*   - Functional activities and walking capacity training tailored to both the patient and his/her home environment. - The functional activities training changes motor, environmental, and cognitive aspects of the tasks, for example, taking different objects from a high shelf. - Trainings were applied at a moderate intensity. - Additional training was suggested for 4 times per week, either on their own or with the help of friends or relatives. - Delivered by physical therapists at the participant’s home. - Supervised sessions: 3 to 6 weeks, 30 min per session, two sessions a week. Moderate intensity. - Unsupervised sessions: 4 times per week. | *Control group*   - Usual care, which was one group session of patient education about the hip surgery operation, walking with crutches, and postoperative exercise from a physical therapist. | *ADLs/IADLs.* None.  *Physical functioning.*   - Chair rise time. - TUG. - 6-minute walk test.   *Cognitive function.* None. | - The *multicomponent home-based preoperative training* group showed greater improvement in the 6-minute walk test than the control group from baseline to prior to admission. Other outcomes were not significantly different. |
| **C2. Multicomponent approach (*n* = 1)** | | | | | |
| van Lieshout 2018 (57)  Netherlands | RCT  Participants were recruited from a semi-rural community.  Functional training group:  *n* = 139; Mean age (SD) = 73.3 (6.7); Female *n* (%) = 82 (59); Drop-out *n* (%) = 47 (33.8).  Control group:  *n* = 142; Mean age (SD) = 74.7 (7.6); Female *n* (%) = 73 (51.4); Drop-out *n* (%) = 27 (19). | *SPRY Program (structured interdisciplinary intervention program)*   - The program included optimization of medication use, improvement of physical fitness, empowerment of social skills, and optimization of nutritional status. - Physical fitness and training in daily activities (walking stairs, shopping, moving outdoors, and standing up from a chair or a bed) to use the interactions between the cognitive, perceptual, motor functions, and the individual’s dynamic environment. - The training was conducted by a physical therapist twice per week for 12 weeks in a local gym. - Group-based, except for the medication component, with an average group-size of 8-10 older adults. - Program was conducted in pharmacy, local gym, and local community center. - 23 weeks, 12 weeks for physical fitness and training in daily activities. | *Control group*   - Care as usual. | *ADLs/IADLs.*   - Katz-6.   *Physical functioning.*   - Physical composite score from the Short Form-12 survey.   *Cognitive function.* None. | - No group difference was found in the ADL performance and the overall physical function at one-year follow up. |

*Note.* a-First author’s last name and publication year. ADL: Activities of daily living. AMPS: Assessment of Motor and Process Skills. CVFT: Category Verbal Fluency Test. CVVLT: Chinese version of the Verbal Learning Test. IADL: Instrumental Activities of Daily Living. LLFDI: Late-Life Function and Disability Instrument. MCI: Mild Cognitive Impairment. MMSE: Mini-Mental State Examination. NCSE: Neurobehavioral Cognitive Status Examination. PEDL: Problems in Everyday Living Test. TMT: Trail-Making Test. TUG: Timed Up and Go Test. VFT: Verbal Fluency Test. VLT: Verbal Learning Test.
