# Supplementary material for: Is functional training functional? a systematic review of its effects in community-dwelling older adults
Source: Eur Rev Aging Phys Act. 2024 Dec 21;21:32. doi: 10.1186/s11556-024-00366-3 (PMC11664925; doi:10.1186/s11556-024-00366-3)
Supplement: Supplementary file 4 — Supplementary Material 4 [file 11556_2024_366_MOESM3_ESM.docx]

Appendix D.

Table 1. List of reviewed outcome measures.

| **Measurement categories** | **Measurement Names (Number of studies: List of Study ID)** |
| --- | --- |
| Activities of Daily Living | |
| Personal activities of daily living | Barthel Index (1: Faria 2023)  Functional Independence Measure (1: Todo 2021)  Katz ADL index (2: Hummer 2015, van Lieshout 2018)  Modified Katiz-15 (1: Siemonsma 2018) |
| Instrumental activities of daily living | Activities of Daily Living Questionnaire (1: Law 2018)  Frenchay Activities Index (1: Comans 2010)  Lawton IADL scale (5: de Freitas 2021, Faria 2023, Law 2013, Law 2014, Liao 2020) |
| Combined personal activities of daily living and instrumental activities of daily living | Assessment of Motor and Process Skills-Motor Skills (3: Liu 2016, Liu 2017, Liu 2020)  Canadian Occupational Performance Measure (1: Liu 2020)  Disability assessment for dementia (1: Harwood 2023)  Groningen Activity Restriction Scale (2: Mohammed 2022, Siemonsma 2018)  Late-life Functional and Disability Instrument (3: Burton 2013, Clemson 2012, Liu 2016)  National Health and Nutrition Examination Survey (NHANES) ADL scale (1: Clemson 2012)  Nottingham Extended ADL Scale (3: Harwood 2023, King 2012, Straubmeier 2017)  Revised Direct Assessment of Functional Status (1: Pedroso 2018) |
| Physical Functioning | |
| Overall physical functioning | Short Form 36 Physical (2: King 2012, van Lieshout 2018)  Senior Fitness Test Battery (3: Barcelona 2023, Faria 2023, Pedroso 2018) |
| Upper body | Box and block test (3: Liu 2016, Liu 2017, Liu 2020)  Functional upper body strength test (1: Solberg 2013)  9-hole peg test (1: Comans 2010) |
| Lower body | GAITRite (2: Halvarsson 2011, Oh 2021)  One-time sit to stand (1: Burton 2013)  Stair climbing (2: Hauer 2012, Solberg 2013)  Step test (1: Comans 2010)  Short Physical Performance Battery (1: Hu 2023)  Timed-Up-Go (14: Burton 2013, Comans 2010, Harwood 2023, Hauer 2012, Karóczi 2014, King 2012, Li 2018, Liu 2017, Liu 2020, Nott 2019, Oh 2021, Oosting 2012, Solberg 2013, Szanton 2021)  Two-minute-step-in-place test (1: Karóczi 2014)  5 times chair stand test (5: Burton 2013, Hauer 2012, Karóczi 2014, Oosting 2012, Solberg 2013)  6-minute walk tests (3: Nott 2019, Oosting 2012, Solberg 2013)  10-meter walking (1: Solberg 2013)  30-second chair stand test (3: Law 2018, Li 2018, Liu 2020) |
| Balance | Ability to stand without assistance (1: Todo 2021)  Berg Balance Scale (3: Harwood 2023, Hu 2023, Oh 2021)  Five-level static balance scale (1: Clemson 2012)  Eight-level static balance scale (1: Clemson 2012)  Fullerton test (1: Karóczi 2014)  Functional reach test (2: Burton 2013, Li 2018)  One-legged stand (1: Li 2018)  Performance Oriented Motor Assessment (1: Hauer 2012)  Tandem stand (1: Szanton 2021)  Tandem walk (2: Burton 2013, Clemson 2012)  Tinetti Balance Scale (2: Faria 2023, Pedroso 2018) |
| Cognitive Function | |
| Global cognitive function | Abbreviated Mental Test Score (1: Comans 2010)  Cambridge Neuropsychological Test Automated Battery (1: Harwood 2023)  Clinical Dementia Rating (1: Pedroso 2018)  Mini Mental State Exam (3: Nott 2019, Pedroso 2018, Straubmeier 2017)  Montreal Cognitive Assessment (2: Harwood 2023, Liao 2020)  Neurobehavioral Cognitive Status Examination (4: de Freitas 2021, Law 2013, Law 2014, Law 2018) |
| Domain-specific cognitive function | Problems in Everyday Living Test (4: de Freitas 2021, Law 2013, Law 2014, Law 2018)  Trail-making test (4: de Freitas 2021, Law 2014, Law 2018, Pedroso 2018)  Verbal fluency test (4: de Freitas 2021, Harwood 2023, Law 2013, Pedroso 2018)  Chinese version of the verbal learning test (3: Law 2013, Law 2014, Liao 2020)  Category verbal fluency test (2: Law 2014, Law 2018)  Verbal learning test (1: de Freitas 2021)  The Executive Interview 25 (1: Liao 2020)  Digit span test (1: Pedroso 2018)  Toulouse-Pieron Concentrated Attention Test (1: Pedroso 2018) |
